# Modeling the Effects of Disease, Drug Properties, and Material on Drug Transport From Intraocular Lenses

**DOI:** 10.1167/tvst.11.5.14

**Published:** 2022-05-16

**Authors:** Danielle P. Clasky, Louise Meunier, Laura A. Wells

**Affiliations:** 1 Department of Chemical Engineering, Queen's University, Kingston, Ontario, Canada; 2 Centre for Health Innovation, Queen's University and Kingston Health Sciences Centre, Kingston, Ontario, Canada

**Keywords:** drug release, computer modeling, intraocular lenses, drug transport, ocular pharmacokinetics

## Abstract

**Purpose:**

Surgically implanted intraocular lenses (IOLs) may be used as drug-delivery devices, but their effectiveness is not well defined. Computational fluid dynamics models were developed to investigate the capability of IOLs to release drugs at therapeutic concentrations.

**Methods:**

Models were generated using COMSOL Multiphysics. Primary open-angle glaucoma (POAG) and wet age-related macular degeneration (AMD) were simulated by reducing aqueous vein and choroidal blood flow, respectively. Release of dexamethasone, ganciclovir, or dextran was studied using common IOL materials, polydimethylsiloxane (PDMS) and poly(2-hydroxyethyl methacrylate) (PHEMA).

**Results:**

Drug clearance proceeds mainly through choroidal blood flow. When fully constricted, maximum concentration at the choroid (*C_max_*) values increased by 32.4% to 39,800%. Compared to dexamethasone, *C_max_* in different tissues decreased by 6.07% to 96.0% for ganciclovir and dextran, and clearance rates decreased by 16% to 69% for ganciclovir and by 92% to 100% for dextran. Using PDMS as the IOL reduced clearance rates by 91.3% to 94.6% compared to PHEMA.

**Conclusions:**

In diseased eyes, drugs accumulate mainly in posterior tissue; thus, choroidal drug toxicity must be assessed prior to IOL implantation in POAG and AMD patients. Moreover, drug properties modulated concentration profiles in all ocular segments. The hydrophobic small-molecule dexamethasone attained the highest concentrations and cleared the fastest, whereas hydrophilic macromolecular dextran attained the lowest concentrations and cleared the slowest. Furthermore, high concentrations were achieved quickly following release from PHEMA, whereas PDMS allowed for sustained release.

**Translational Relevance:**

In silico results can guide scientists and clinicians regarding important physiological and chemical factors that modulate tissue drug concentrations from drug-eluting IOLs.

## Introduction

Cataracts are the leading cause of blindness worldwide and affect approximately 21 million American adults.^1^ A cataractous lens is removed through phacoemulsification and replaced by an intraocular lens (IOL).^1^ Despite the positive outcomes of cataract surgery, complications may arise, including posterior capsule opacification (PCO),^2^ especially for patients with secondary diseases such as uveitis. PCO is typically treated by removing the posterior capsule by neodymium-doped yttrium aluminum garnet (Nd:YAG) laser capsulotomy, which is inconvenient and costly.^3^ Additionally, uveitis may arise post-surgery, leading to vision reduction or loss.^4^ However, drug treatments such as corticosteroids can reduce inflammation caused by uveitis, and recent evidence suggests that similar drugs could reduce the occurrence of PCO.^5^^–^^7^

To treat PCO and other ailments in the eye, drugs must be delivered at effective concentrations over the proper period. Conventional administration techniques include topical eye drops and periocular/intravitreal injections.^8^ Low bioavailability is observed following topical administration and periocular injections, and intravitreal injections have low patient compliance and may lead to endophthalmitis, retinal detachment, and/or hemorrhage.^8^ Such challenges prompted the development of alternative approaches. Drug-loaded implants are an attractive option because they can be placed near damaged tissue and be designed for sustained release.^8^

Because IOLs are implanted during cataract surgery, they may be used as drug-delivery devices to prevent, alleviate, and treat post-surgery complications.^3^ Drug can be preloaded into IOLs and released following implantation. Before an IOL may be used in this manner, tests must be performed to determine whether it can release drugs at therapeutic yet nontoxic concentrations to appropriate eye segments. True concentration measurements require human trials, but these cannot be conducted until estimates are obtained and laboratory tests are conducted.

Such estimates may be obtained through in silico modeling. Ocular drug delivery studies have been performed using COMSOL (Stockholm, Sweden) Multiphysics,^9^ a computational fluid dynamics (CFD) tool that offers full simulation capability incorporating fluid mechanics, heat transfer, and transport phenomena. Studies have explored topical drug administration^10^^,^^11^ and release from episcleral and subconjunctival implants,^12^ but no existing models simulate release from an IOL. Also, previous models do not simulate diseased conditions known to alter the physical properties of ocular tissues. By altering choroidal and aqueous blood flow rates, the effects of conditions such as primary open-angle glaucoma (POAG) and wet age-related macular degeneration (AMD) on drug clearance or accumulation can be predicted.

In the present research, CFD models were generated to simulate and quantify physiologically representative drug release from an IOL in both healthy and diseased eyes. Three drugs with varying properties were assessed: dexamethasone (hydrophobic small-molecule drug), ganciclovir (hydrophilic small-molecule drug), and dextran (hydrophilic macromolecular model drug). CFD models were used to obtain estimates of drug concentration ranges within ocular tissue. Estimates aid in determining whether an IOL can effectively release a given drug to treat post-cataract surgery complications. Specifically, modeling can characterize drug concentration ranges, accumulation, and clearance rates in various eye segments, as well as release rates from different IOL materials.

## Methods

CFD models were designed to mimic the physiology of a healthy adult human eye and then used to simulate drug release from an IOL. Models were generated using COMSOL Multiphysics 5.6^9^ and included interfaces based on heat transfer (HT), laminar flow (LF), Darcy's law (DL) (flow through porous media), and transport of diluted species (TDS), which account for equations of motion and transport.^9^ Models were built in increments, starting with constant flow and temperatures before incorporating more representative and physiologically relevant parameters. In the second generation of models, steady-state temperature and flow profiles were generated for the eye geometry, and then, in the fully developed models, drug transport was studied under various conditions. In addition to the descriptions presented in this manuscript, details of modeling equations, parameters, and methodology are presented in the Supplementary Materials.

### Physiologically Representative CFD Models

Determining accurate anatomical and physiological features of adult eyes, IOL properties, and ocular drug transport mechanisms is critical for developing representative models. When the environment has been established, properties may be varied within the CFD models to modulate drug release. The eye is roughly spherical, with a diameter between 23 and 26 mm.^1^ It is divided into the anterior (front of eye to lens front) and posterior (space behind the lens) segments.^1^ The anterior is filled with a clear fluid (aqueous humor), and it also contains the iris and ciliary body.^1^ The cornea encloses the aqueous humor at the front of the eye. The posterior segment is filled with a gel-like liquid (vitreous humor), which is surrounded by successive layers: the retina, choroid, and sclera.^1^

Ocular heat transfer and fluid flow affect drug mass transport and result in a temperature profile.^8^ Heat is transferred from blood in the ophthalmic artery to the sclera by convection and through the aqueous and vitreous humor by convection; heat is lost from the cornea to the environment through convection, radiation, and tear evaporation.^13^^,^^14^ Subdomain dimensions and material properties are described in the Supplementary Materials, Sections B and C.

Fluid flow occurs in both anterior and posterior segments. Aqueous humor enters the anterior segment from the ciliary body (where it is produced) with a flow rate of approximately 2.4 µL/min. Aqueous humor exits via the conventional route, moving through the trabecular meshwork and draining into the Schlemm's canal, or via the uveoscleral or uveovortex pathway (unconventional routes).^15^ Only the conventional route was included in the CFD models because little is known about the contribution of unconventional routes to aqueous humor outflow due to the complexities of flow measurement.^16^ Fluid flow through the posterior segment results from posterior-directed aqueous flow. The vitreous humor may thus be considered a porous medium capable of transmitting fluids.^17^^,^^18^

Mass transfer through ocular tissue results from passive diffusion, convection, and active transport.^19^ The driving force for passive diffusion is the concentration gradient. Diffusion rates depend on drug physiochemical properties, such as hydrophilicity/hydrophobicity, molecular weight, molecular radius, and charge.^20^ The diffusion coefficient of a drug describes its rate and ease of diffusion through a given ocular medium. Convection refers to the transport of a species by bulk flow,^21^ which occurs in both aqueous and vitreous humors. To undergo active transport across cell membranes, drugs must bind to cell receptors and be transported across cellular barriers by carrier proteins.^19^ Whether active transport significantly affects ocular pharmacokinetics is unclear because transporter expression levels are not well characterized.^19^ Therefore, only passive diffusion and convection were incorporated in the CFD models.

Drugs are eliminated from the anterior segment by the draining of fluids into the Schlemm's canal and then the aqueous vein, or by diffusing through the ciliary body into the choroid.^20^ Posterior elimination follows drug diffusion through the retina into the choroid.^20^ When they have entered the choroid, drugs either diffuse into the sclera and then surrounding tissue or are rapidly cleared by blood flow.^20^ In the CFD models, the elimination of drug was accounted for by incorporating choroidal and aqueous vein blood flows and by drug diffusion from the sclera into the surrounding bulk fluid.

### Intraocular Lenses for the CFD Model

IOLs are typically 5 to 6 mm in diameter, with a thickness of 1 to 3 mm.^22^ IOLs are made of polymer biomaterials that are classified as either hydrophobic or hydrophilic.^1^ Materials include silicones, polydimethylsiloxane (PDMS), and acrylic copolymers made of methyl methacrylate, 2-hydroxyethyl methacrylate, and/or 2-phenylethyl acrylate.^23^ Two materials were modeled: PDMS (hydrophobic) and poly(2-hydroxyethyl methacrylate), or PHEMA (hydrophilic). Drug loading into IOLs affects drug release. The models consider drugs that are incorporated into an IOL after synthesis by incubation in high-concentration drug solutions.^24^ Therefore, the initial IOL concentrations were selected based on drug solubility (details in Supplementary Materials, Section D) and from which diffusion coefficients of drugs through IOL materials were estimated (details in Supplementary Materials, Sections E and F).

### Eye Geometry

**Figure 1.**
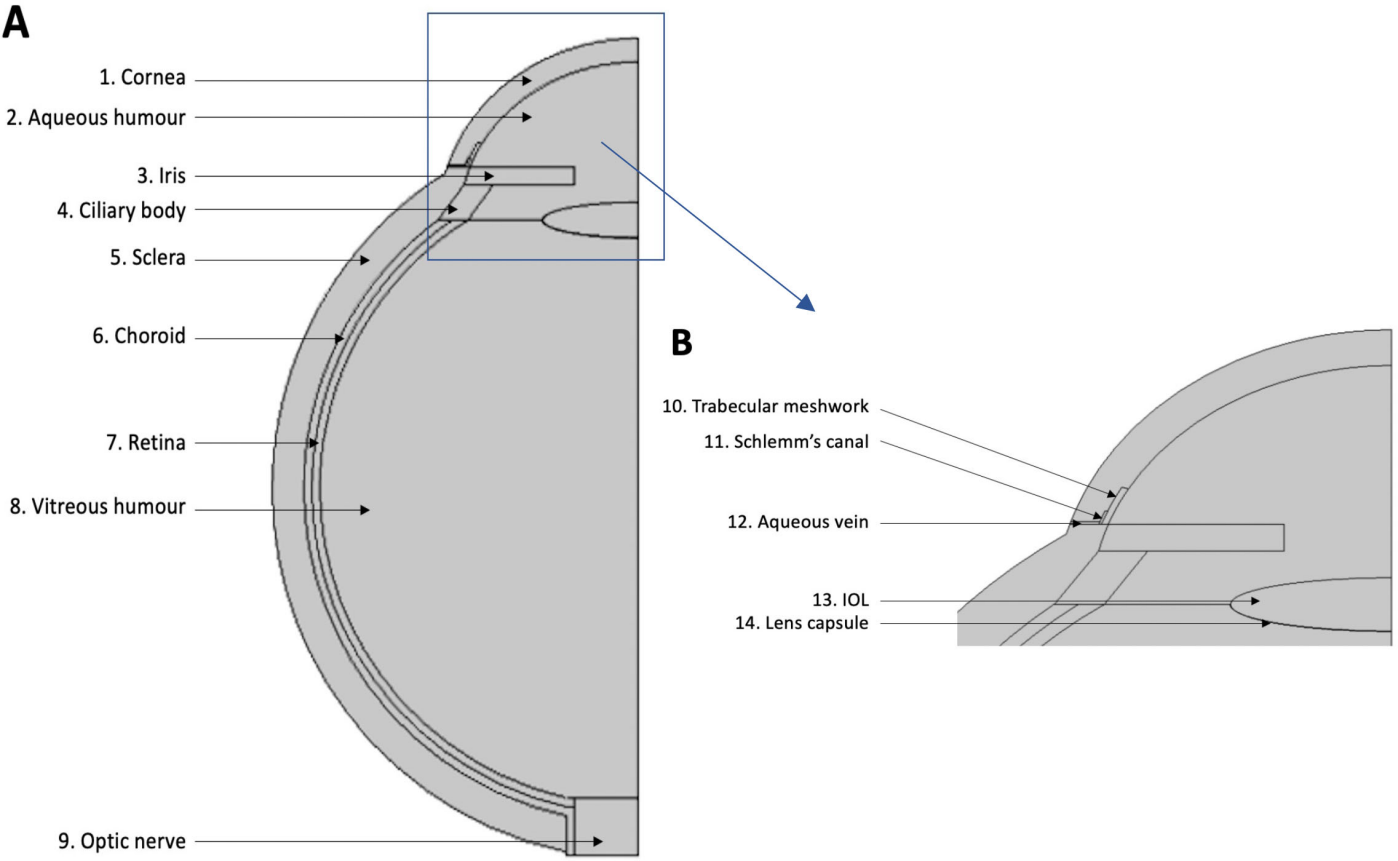
Eye geometry with labeled subdomains. (**A**) Entire geometry (*top view*). (**B**) Details of the contents of the *blue box* in image A. Dimensions used to build geometry and subdomain material properties were obtained from the literature (values are listed in Supplementary Materials, Sections B and C).

The model consists of 14 subdomains, shown in Figure 1. Dimensions and material properties are listed in Supplementary Materials, Sections B and C, respectively. CFD models feature an axis of symmetry; thus, only half of the eye geometry is shown. The assumption of symmetry along the central axis of the eye does not constrict the modeling flow itself but rather serves to reduce computational time with a mirror image. A zero net flux boundary condition is set at the axis of symmetry.

### Steady-State Ocular Profiles

**Figure 2.**
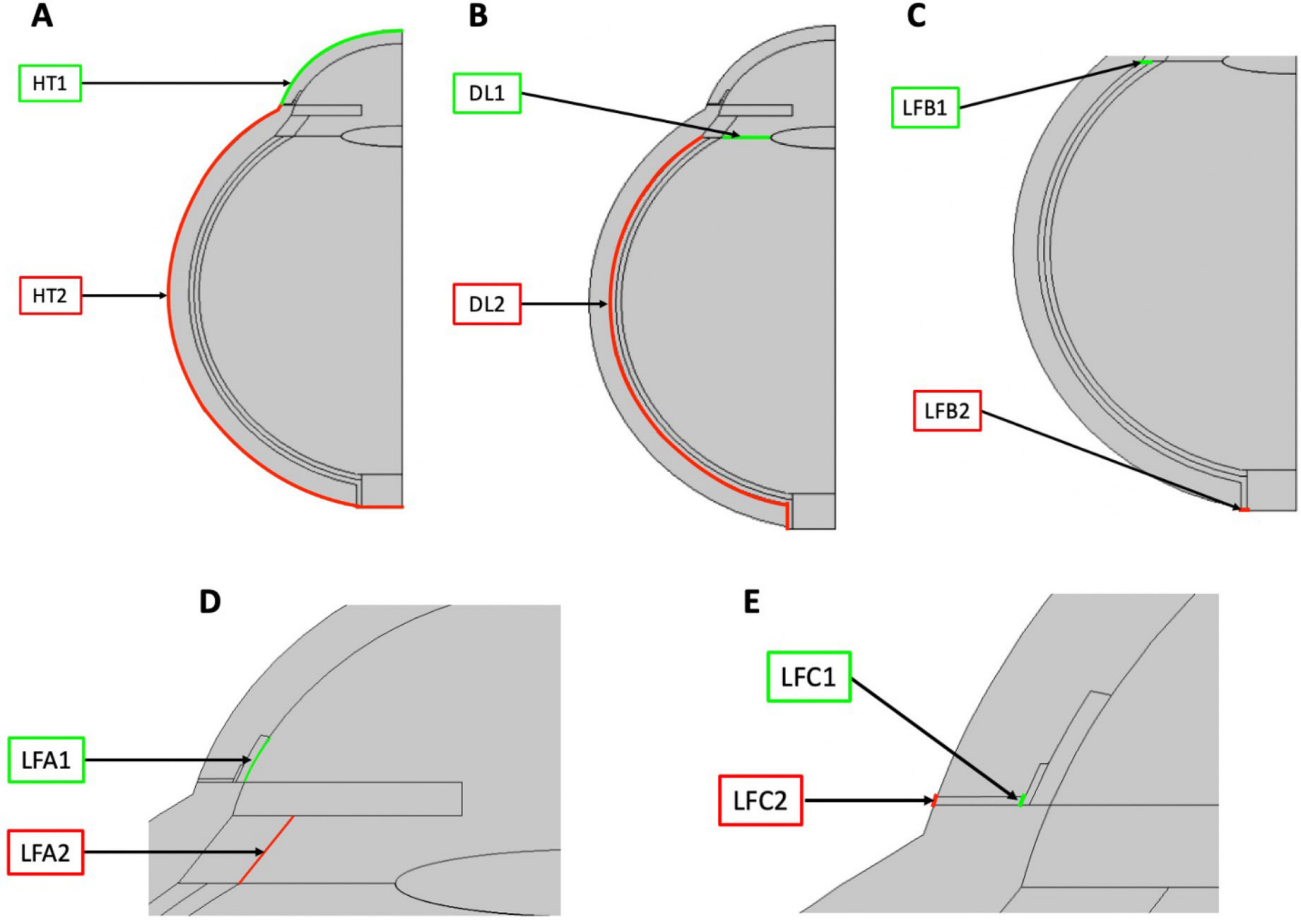
Boundary conditions for each physical interface. (**A**) The HT interface was used to generate the temperature profile. (**B**) The DL interface was used to generate the vitreous flow profile. (**C**) The DL and LFB interfaces were used to generate the choroidal blood flow profile. (**D**) The DL and LFA interfaces were used to generate the aqueous flow profile. (**E**) The DL and LFC interfaces were used to generate the aqueous vein blood flow profile.

Steady-state temperature and flow profiles establish the physical properties of the model. Temperature profiles were generated from the temperature of blood and air by applying the HT interface to all subdomains. The DL interface was applied to subdomains 6, 7, and 8 to obtain the flow profile of the vitreous humor. The LF interface was applied to subdomain 2 to obtain the flow profile of the aqueous humor (LFA). Elimination was incorporated by adding the LF interface to subdomain 6 (LFB) and subdomain 12 (LFC) to simulate choroidal and aqueous vein blood flows. Boundary conditions applied to each physical interface are labeled in Figure 2 and are described in Tables 1 and 2. A condition of no net flux was applied at the centerline in all cases, representing symmetry.

**Table 1.** Descriptions of Boundary Conditions Used To Generate Steady-State Temperature Profile

Boundary Condition	Description	Expression^a^	Values^b^
HT1^12^^,^^25^^,^^26^	Convection with ambient air	*q* = *h*_1_(*T* – *T_a_*)	*h*_1_ = 10 W/m^2^**·**K *T_a_* = 298 K
	Radiation with ambient air	$q=\sigma ɛ(T^{4}-T_{a}^{4})$	σ = 5.67 × 10^−8^ W/m^2^**·**K^4^ ε = 0.975 *T_a_* = 298 K
	Tear evaporation	*q* = *E*	*T* = 40 W/m^2^
HT2^12^^,^^25^^,^^26^	Convection with blood	*q* = *h*_2_(*T* – *T_bl_*)	*h*_2_ = 65 W/m^2^**·**K *T_bl_* = 310 K

a *q* is the heat flux (W/m^2^), *h*_1_ is the convective heat transfer coefficient of the cornea (W/m^2^**·**K), *T* is the time-/space-dependent model temperature (K), *T_a_* is the ambient temperature (K), σ is the Stefan–Boltzmann constant (W/m^2^**·**K^4^), ε is the corneal surface emissivity, *E* is the tear evaporation rate (W/m^2^), *h*_2_ is the convective heat transfer coefficient of the sclera (W/m^2^**·**K), and *T_bl_* is the blood temperature (K).

b Input parameters are provided. Heat fluxes (*q*) were calculated during simulation using model temperature (*T*).

**Table 2.** Descriptions of Boundary Conditions Used To Generate Steady-State Flow Profiles

Boundary Condition	Description	Value
DL1^18^^,^^27^	Pressure at anterior vitreous	2000 Pa^a^
DL2^18^^,^^27^	Pressure at posterior choroid	1300 Pa^a^
LFA1^12^	Pressure at trabecular meshwork	2000 Pa
LFA2^b^	Aqueous humor velocity at ciliary body	1.14 × 10^−6^ m/s
LFB1^12^	Pressure at choroid top	2000 Pa
LFB2^28^	Blood velocity at choroid bottom	6.00 × 10^−3^ m/s
LFC1^c^	Blood velocity at aqueous vein inlet	9.17 × 10^−3^ m/s
LFC2^29^	Pressure at aqueous vein outlet	1067 Pa

a Pressure is shown as approximately constant in DL1 and DL2 and transitions gradually, element by element, between modeled segments such that instantaneous flow rates remain small (details in Supplementary Materials, Section C).

b Velocity is calculated by dividing the aqueous humor volumetric flow rate (2.4 µL/min) by the surface area of the ciliary body (35 mm^2^).^12^

c Velocity is calculated by dividing the volumetric blood flow rate in the aqueous vein (1.08 µL/min) by the surface area of the aqueous vein (circle with diameter of 50 µm).^30^

### Model Inputs and Boundary Conditions for Time-Dependent Drug Transport

**Figure 3.**
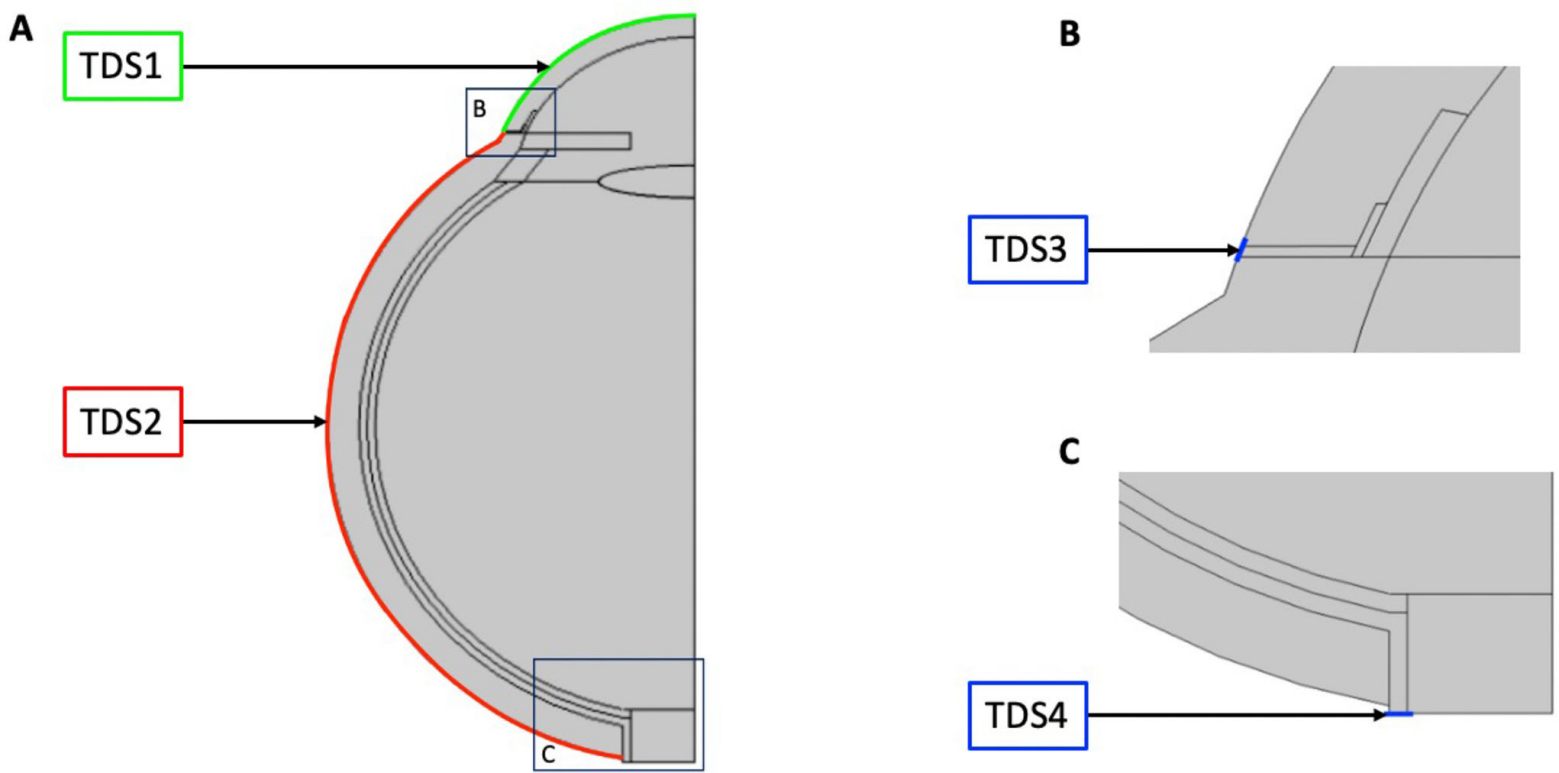
Boundary conditions for the TDS interface. (**A**) Entire geometry. (**B**) Details of the top *blue box* in image A. (**C**) Details of the bottom *blue box* in image A.

Drug transport was incorporated by applying the TDS interface to all subdomains except the optic nerve (subdomain 9). The optic nerve is assumed impermeable to drugs because it is impermeable to tracer molecules.^31^^–^^33^ TDS interface inputs included the initial IOL concentration in subdomain 13 and drug diffusion coefficients in every subdomain. An initial IOL concentration of 0.20 mol/m^3^ was based on drug solubility (see Supplementary Materials, Section D). Drug diffusion coefficients through ocular tissue and the IOL are listed in Supplementary Materials, Sections E and F, respectively. Boundary conditions are displayed in Figure 3 and are described in Table 3. A condition of no flux was applied at the centerline, representing symmetry.

**Table 3.** Descriptions of Boundary Conditions Used To Study Drug Transport

Boundary Condition	Description	Expression^a^	Values^b^
TDS1^12^^,^^25^^,^^26^	No net flux	*N* = 0	N/A
TDS2^12^^,^^25^^,^^26^	External convection with blood	*N* = *k_s_* (*C* – *C_b_*), where *k_s_* = *D_ij_*/*x*	*D_ij_* = species- and material-dependent^c^*x* = 0.001 m*C_b_* = 0 mol/m^3^
TDS3 and TDS4^d^	Outflow	*N* = *UCJ* = 0^e^	N/A

a *N* is the total molar flux (mol/m^2^**·**s); *k_s_* is the mass transfer coefficient of the sclera (m/s); *C* is the time-/space-dependent model drug concentration (mol/m^3^); *C_b_* is the bulk drug concentration in surrounding blood (mol/m^3^); *D_ij_* is the diffusion coefficient of species *i* in material or tissue *j* (m^2^/s), with values listed in Supplementary Table SE-1; *x* is the thickness of the sclera (m); *U* is the medium flow velocity (m/s); and *J* is the diffusive molar flux (mol/m^2^**·**s).

b Input parameters are provided. Molar fluxes (*N*) were calculated during simulation using model concentration (*C*) and flow velocity (*U*).

c The diffusion coefficient in each medium varies based on species (values are provided in Supplementary Materials, Sections E and F).

d Previous models did not account for elimination mechanisms and thus did not employ these boundary conditions.

e The outflow boundary condition assumes that convection is the dominant transport mechanism across the boundary, so diffusive transport is neglected.^9^

### Time-Dependent Studies Exploring Changes in Physiological and Transport Parameters

Time-dependent drug release from IOL was estimated through successive studies. First, the base case characterized normal physiological conditions (all steady-state interfaces were enabled so all elimination routes were active), and dexamethasone release from a PHEMA IOL was analyzed. Details pertaining to the drug, IOL material, and steady-state interfaces utilized in each study are provided in the Supplementary Materials, Section G. This base case was used as a comparison for all other studies.

Blood flow was reduced to model the effects of disease. Aqueous vein blood flow was removed by disabling the LFC interface, and choroidal blood flow was removed by disabling the LFB interface. Decreased clearance via aqueous veins is characteristic of POAG,^34^ and decreased choroidal flow is observed in wet AMD.^35^ Elimination mechanism deactivation was a starting point for modeling POAG and wet AMD by simulating extreme cases. Afterward, varying disease severity was simulated by reducing the flow rate of blood through the choroid (subdomain 6) by 50% to 99% of its initial value to examine the relationship between choroidal blood flow and drug transport.

Three drugs were analyzed to assess the effects of drug properties on drug transport. Dexamethasone is a hydrophobic small molecule (octanol/water partition coefficient is 68,^36^ 392 Da^37^); ganciclovir is a hydrophilic small molecule (octanol/water partition coefficient is 0.022,^38^ 255 Da^37^); and dextran is a hydrophilic macromolecule (octanol/water partition coefficient is 0.33,^39^ 40,000 Da^37^). Additional drug properties are listed in the Supplementary Materials, Section H. Finally, the IOL material characteristics were altered from PHEMA to PDMS by changing the drug diffusion coefficient in subdomain 13 (the IOL) to that of dexamethasone in PDMS (values in Supplementary Materials, Section F).

Study results included maximum concentrations of drug in different ocular segments (*C_max_*), clearance rates from the tissues (*C_l_*), and times for maximum concentrations to be reduced by one half (*t_h_*). Results from the different studies were compared to the normal physiologic conditions (base case) by calculating percent changes. Further details pertaining to data analysis are provided in the Supplementary Materials, Section I. Changes were considered observable when above a 5% threshold and were further classified according to their magnitude: class 1 (5%–100%), class 2 (100%–1000%), and class 3 (>1000%). Any changes below 5% were attributed to expected deviations within the modeling software and not considered physiologically representative.

## Results

CFD models were generated as a tool to characterize the effects of disease, drug properties, and IOL material on drug concentration ranges in ocular tissue following IOL release. First, steady-state ocular temperature and flow profiles were generated because these phenomena affect drug transport and clearance and thus are necessary to obtain accurate drug concentration profiles. Time-dependent studies were then performed to analyze the effects of diseased conditions, drug properties, and IOL material on drug concentration ranges within ocular tissue.

### Steady-State Ocular Profiles Represent Physiological Conditions

**Figure 4.**
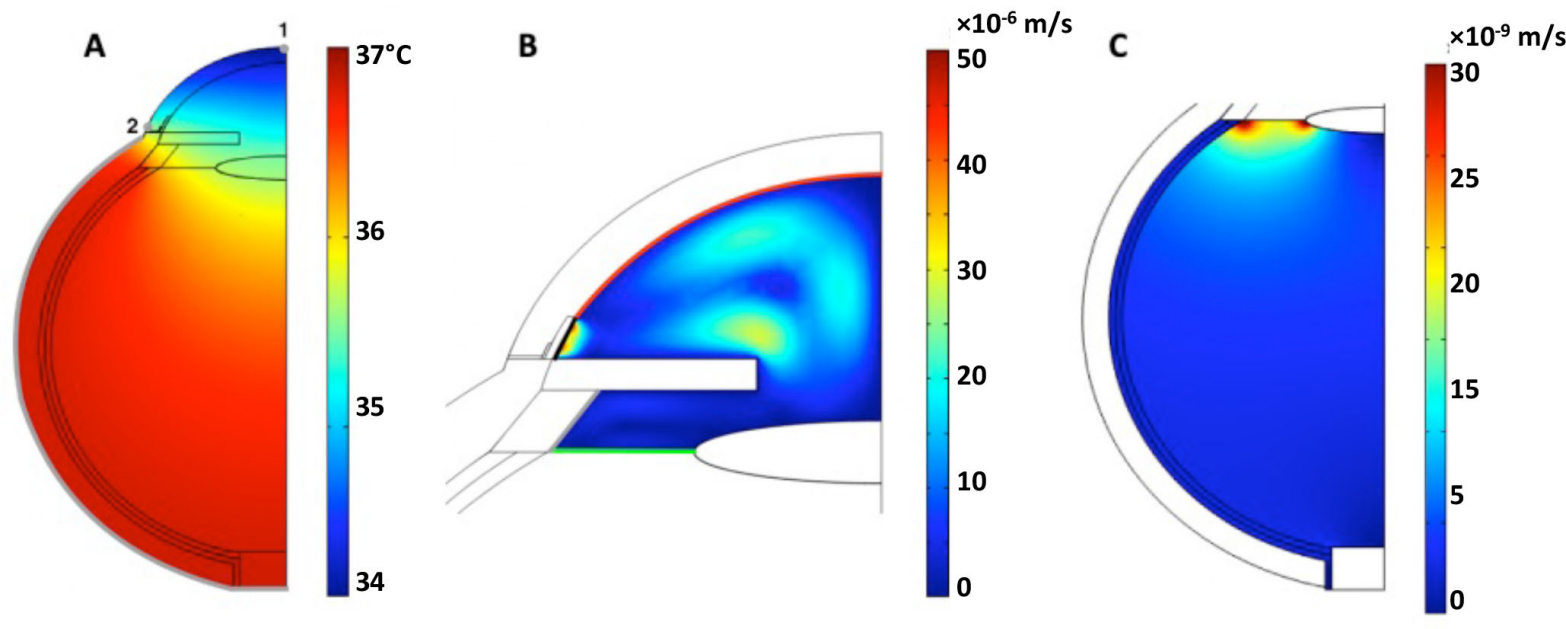
(**A**) Temperature profile, (**B**) aqueous flow profile, and (**C**) vitreous flow profile. In (**A**), the legend represents temperature (°C). In (**B**) and (**C**), the legend represents flow velocity (m/s). In (**A**), point 1 is the central cornea (34.5°C), point 2 is the corneal limbus (35.5°C), and the *gray* *boundary* is the sclera outer surface (36.5°C). In (**B**), the aqueous/vitreous (*green*) boundary attains the maximum subdomain temperature of 36.1°C, and the aqueous/cornea (*red*) boundary attains the minimum subdomain temperature of 34.6°C; the *gray* and *black boundaries* are the aqueous humor points of entry and exit, respectively.

Convective drug transport is a result of aqueous and vitreous flow, and these flow profiles depend on the ocular temperature gradient. These temperature differences may also promote buoyancy-driven aqueous flow or vitreous flow within the eye which can change during different positioning or be altered when eyes are closed for periods of time. The model here does not include changes in buoyancy-driven flow due to thermal expansion with different positioning of the eye to enable direct comparisons between difference scenarios, similar to previous works such as that by Stay et al.^40^ The flow velocity profiles shown in Figure 4 resulted from these convective movements, as depicted in a two-dimensional vertical slice through the central axis of the eye. Many previous studies predicting the distribution of drugs within the eye have focused on bolus injections into the vitreous. The distribution of drug in these studies is highly influenced by buoyancy because of large differences in the density of the fluid being injected in comparison to the vitreous.^41^ The scenario here is quite different; the drugs are modeled to diffuse into the vitreous from a separate compartment (anterior side of the posterior capsule). Convective movements result from temperature gradients rather than from any variations in fluid density, which is nearly constant within each subdomain (values are listed in Supplementary Materials, Section C). Accordingly, temperature and flow profiles (Fig. 4) were generated prior to analysis of drug transport.

Ocular temperature increased by 2.5°C from the front to the back of the eye. The central surface corneal temperature was 34.5°C (point 1 in Fig. 4A), whereas the limbus surface corneal temperature was 35.5°C (point 2 in Fig. 4A). The average temperature at the sclera outer surface was 36.5°C (gray boundary in Fig. 4A) due to its proximity to the ophthalmic artery, which was modeled to contain blood at 37°C. These results match previously reported temperatures^42^ (details in Supplementary Materials, Section J). Earlier models were generated to consider only constant-temperature domains, but these were not retained because they neglect these convective contributions to fluid flow. Average aqueous and vitreous velocities were 9.63 × 10^−6^ m/s and 6.47 × 10^−9^ m/s, respectively (details in Supplementary Materials, Section J). These results confirm previously published predictions, such as those by Xu et al.^18^ and Shafahi and Vafai,^43^ that convection does not contribute significantly to transport in the eye, especially when considering low-molecular-weight drugs.

The flow profile shown in Figure 4C is similar to the flow direction distribution in the vitreous depicted in a previous study by Stay et al.^40^ in the initial stage of drug diffusion. However, the Stay et al.^40^ model predicts a continued increase in concentration. Following a concentration peak, the currently proposed model shows a decreased concentration, which is in line with measurements taken in vivo by Normand et al.^44^ In Figures 4B and 4C, leakage is not visible because it is relatively small compared to the bulk flow, which moves from greater to lower pressure regions. Aqueous vein and choroidal blood flow contributes to drug elimination and thus affects drug concentration profiles. Maximum blood velocities were attained at the center of the vessels (1.51 × 10^−2^ m/s for the aqueous vein, 8.68 × 10^−3^ m/s for the choroid) and wall velocities were zero (details in Supplementary Materials, Section J).

### Base Case Results Match Physiologically Representative Steady-State Profiles

**Figure 5.**
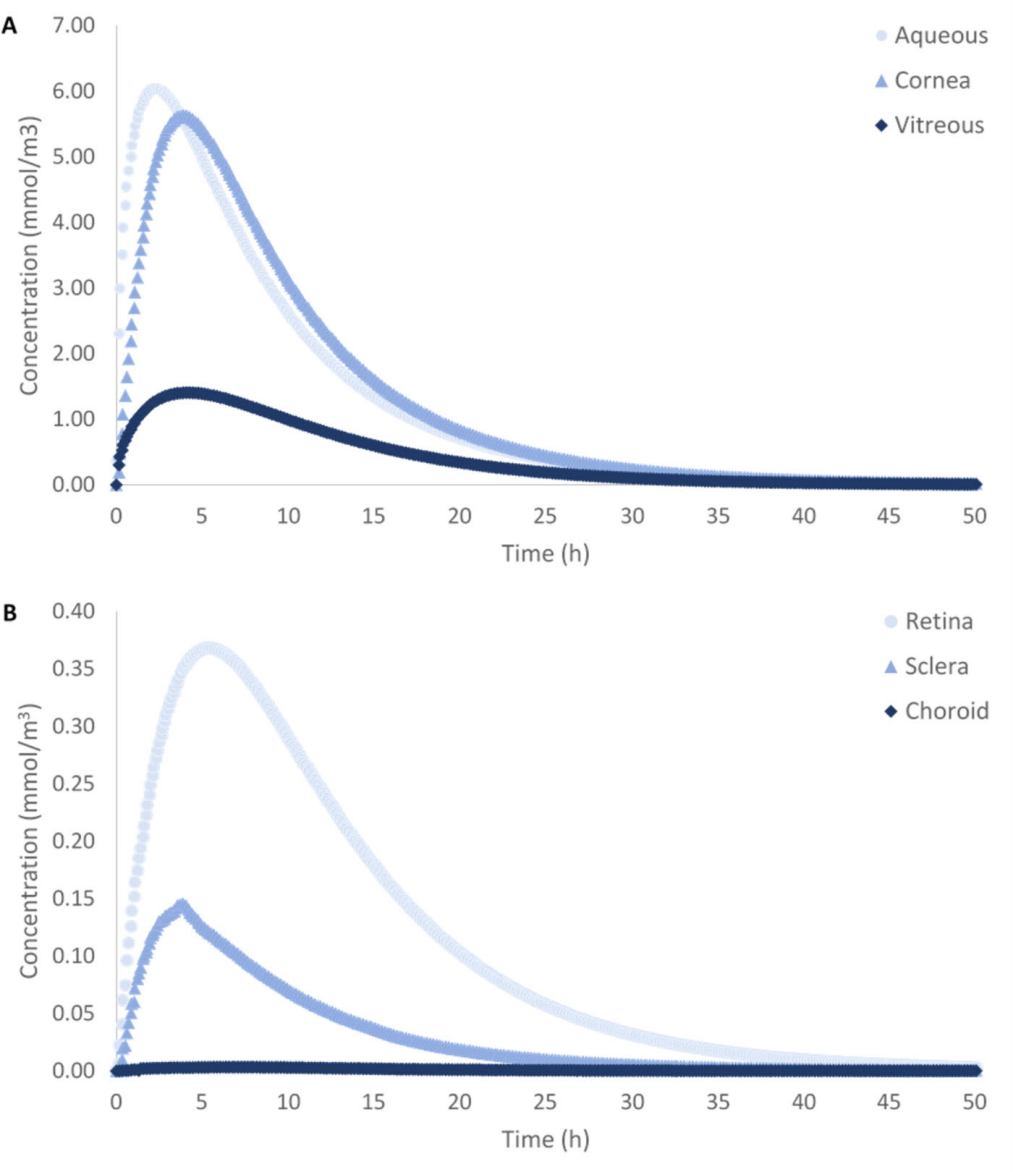
Average drug concentration in the aqueous, cornea, and vitreous (A) and in the retina, sclera, and choroid (B) over a 50-hour period. Maximum concentration (*C_max_*) is represented by the peak on each curve. *C_max_* was greater in anterior tissue (aqueous, cornea) than posterior tissue (vitreous, retina, choroid, and sclera). In the anterior segment, *C_max_* decreased from 6.04 × 10^−3^ mol/m^3^ to 5.63 × 10^−3^ mol/m^3^ with increasing distance from the IOL. In the posterior segment, *C_max_* decreased from 1.41 × 10^−3^ mol/m^3^ to 3.35 × 10^−6^ mol/m^3^ with increasing distance from the IOL (exception of trend in choroid and sclera).

Following dexamethasone release, the concentration in each ocular segment increased until a maximum was attained and then subsequently decreased (concentration patterns are shown in Fig. 5). Details for each ocular segment are reported in the Supplementary Materials, Section K. The average *C_max_* in anterior tissues was 5.84 × 10^−3^ mol/m^3^, whereas it was 4.81 × 10^−4^ mol/m^3^ in posterior tissues. Anterior concentrations were higher because the aqueous flow velocity was three orders of magnitude greater than vitreous flow velocity (details in Supplementary Materials, Section J). Convective transport contributions were thus greater in the anterior segment, leading to greater drug accumulation.

In the posterior segment, *C_max_* varied between 1.41 × 10^−3^ mol/m^3^ and 3.35 × 10^−6^ mol/m^3^. *C_max_* generally decreased with increasing distance from the IOL, except in the choroid and sclera; *C_max_* was lower in the choroid than in the sclera because choroidal blood flow quickly cleared the drug. These profiles align with in vivo studies by Normand et al.,^44^ who tracked intravitreal injections of fluorescent dyes and drugs in non-human primates microscopically over time. They found that the fluorescent intensity of dye in the retina peaked at approximately the same time frame as that shown in Figure 5B.

### Choroidal Flow Is the Main Contributor to Drug Clearance Following IOL Release

*C_max_*, *t_max_*, and *t_h_* values for each ocular segment for studies in which blood flow was eliminated are listed in the Supplementary Materials, Section K. Percent changes in *C_max_* and *t_h_* compared to the base case (normal conditions) are reported in Table 4. Aqueous vein blood flow was stopped to model an extreme case of POAG. Drug could not be eliminated anteriorly and then diffused into the posterior segment to be cleared by choroidal blood flow or diffusion through the sclera. *C_max_* increased by 957% in the choroid and 365% in the sclera as a result. Subsequent elimination from these segments was more rapid than in the base case, as indicated by the 65.0% decrease in *t_h_* for the sclera and the 76.8% decrease in *t_h_* for the choroid. Clearance was faster because the increases in *C_max_* led to higher concentration gradients, which are the driving force for diffusion.

**Table 4.** Percent Change in *C_max_* and *t_h_* for Dexamethasone Without Blood Flow Compared to the Base Case


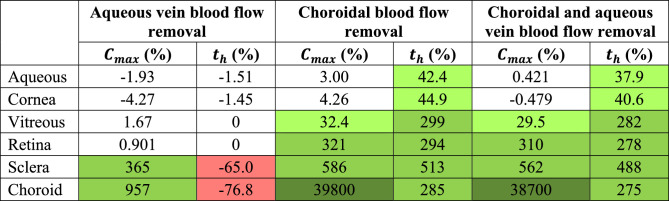

Decreases greater than 5% are shown in red. Class 1 (5%–100%), class 2 (100%–1000%), and class 3 (>1000%) increases are shown in light, medium, and dark green, respectively.

When choroidal blood flow was removed to model an extreme case of wet AMD, increases in *C_max_* in the posterior segment surpassed the 5% threshold. A particularly high increase (class 3) was observed in the choroid; low drug concentration was maintained in the choroid due to rapid clearance by blood flow, and in the absence of this flow the drug could accumulate.

Removal of both elimination mechanisms resulted in trends similar to those observed when choroidal blood flow was removed, and changes in *C_max_* and *t_h_* did not exceed the 5% threshold (details in Supplementary Materials, Section K). This demonstrates that concentration profiles are similar whether or not drug is eliminated via aqueous veins and that choroidal blood flow is the main contributor to clearance following IOL release.

### Choroidal Blood Flow Contributes to Drug Clearance at Low Velocities

Studies reducing (but not eliminating) choroidal blood flow were conducted to model wet AMD cases of varying severity. Inlet velocities are listed and *C_max_*, *t_max_*, and *t_h_* for each ocular segment are reported in the Supplementary Materials, Section L. Percent changes in *C_max_* and *t_h_* compared to the base case are reported in Table 5. Percent changes for blood flow removal (100% reduction) are included in Table 5 for comparison.

**Table 5.** Percent Change in *C_max_* and *t_h_* for Dexamethasone With Reduced Choroidal Flow Compared to the Base Case


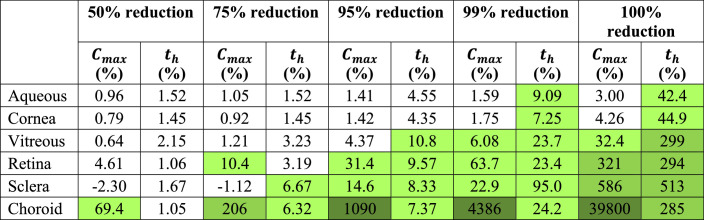

Class 1 (5%–100%), class 2 (100%–1000%), and class 3 (>1000%) increases are shown in light, medium, and dark green, respectively. No decreases met the 5% threshold.

Similar to results obtained from eliminating blood flow, increases in *C_max_* in the aqueous and cornea did not exceed the 5% threshold when blood flow was reduced to a lower degree. *C_max_* surpassed the threshold in the choroid at 50% reduction, but increases greater than 5% in all posterior segments were only observed when flow had been reduced by 99%. The first increases greater than 5% in *t_h_* were apparent at 75% reduction (for the sclera and choroid), but 99% reduction was required for increases in *t_h_* to exceed the threshold in all segments. Additionally, at 99% flow reduction, all increases were class 1, other than the increase in *C_max_* in the choroid (which was class 3), whereas at 100% reduction only increases in *t_h_* in the anterior segment were class 1, and remaining increases were classes 2 or 3 (for *C_max_* in the choroid). This finding implies that choroidal blood flow greatly contributes to drug clearance even at low velocities.

### Hydrophilicity/Hydrophobicity and Molecular Size Modulate Maximum Drug Concentration and Clearance

*C_max_*, *t_max_*, and *C_l_* values for each drug in each ocular segment are reported in Table 6. *C_l_* was calculated between the elimination phase initiation and the time at which IOL concentration was reduced to 0.35% of its initial value (7 × 10^−4^ mol/m^3^). *C_f_* and *t_f_* values utilized in *C_l_* calculations are listed in the Supplementary Materials, Section M. The effects of drug properties on *C_max_* and *C_l_* were assessed by calculating changes for ganciclovir and dextran compared to those for dexamethasone. For ganciclovir compared to dexamethasone, the change in aqueous *C_max_* did not exceed 5%, whereas *C_max_* in other segments decreased by 6.07% to 62.1% (least in the vitreous, most in the sclera). For dextran compared to dexamethasone, the change in vitreous *C_max_* did not exceed 5%, whereas *C_max_* in the retina increased by 111% and decreased by 13.6% to 96.0% in other segments (least in the aqueous, most in the choroid). *C_l_* decreased by 15.9% to 69.7% for ganciclovir compared to dexamethasone and by 92.5% to 99.7% for dextran compared to dexamethasone. Decreases were greatest in the choroid for both drugs and least in the aqueous for ganciclovir and the retina for dextran.

**Table 6.** *C_max_*, *t_max_*, and *C_l_* of the Three Model Drugs in All Ocular Segments

	Dexamethasone			Ganciclovir			Dextran		
	*C_max_* (×10^3^ mol/m^3^)	*t_max_* (h)	*C_l_* (×10^6^ mol/m^3^·h)	*C_max_* (×10^3^ mol/m^3^)	*t_max_* (h)	*C_l_* (×10^6^ mol/m^3^·h)	*C_max_* (×10^3^ mol/m^3^)	*t_max_* (h)	*C_l_* (×10^6^ mol/m^3^·h)
Aqueous	6.04	2.3	286	6.15	2.0	241	5.22	2.8	19.1
Cornea	5.63	3.9	283	4.39	5.1	182	2.53	21.0	8.64
Vitreous	1.41	4.2	65.1	1.32	4.5	45.4	1.44	22.2	2.39
Retina	0.368	5.4	17.6	0.315	7.9	11.3	0.776	58.7	1.32
Sclera	0.146	3.8	7.42	0.0551	6.3	2.31	0.0564	13.1	0.196
Choroid	0.00335	6.7	0.165	0.00154	7.9	0.0501	0.000133	83.0	0.000521

*C_l_* was calculated from elimination phase initiation to the time at which IOL concentration was reduced to 0.35% of its initial value.

### Concentration Profiles Vary with IOL Material

**Figure 6.**
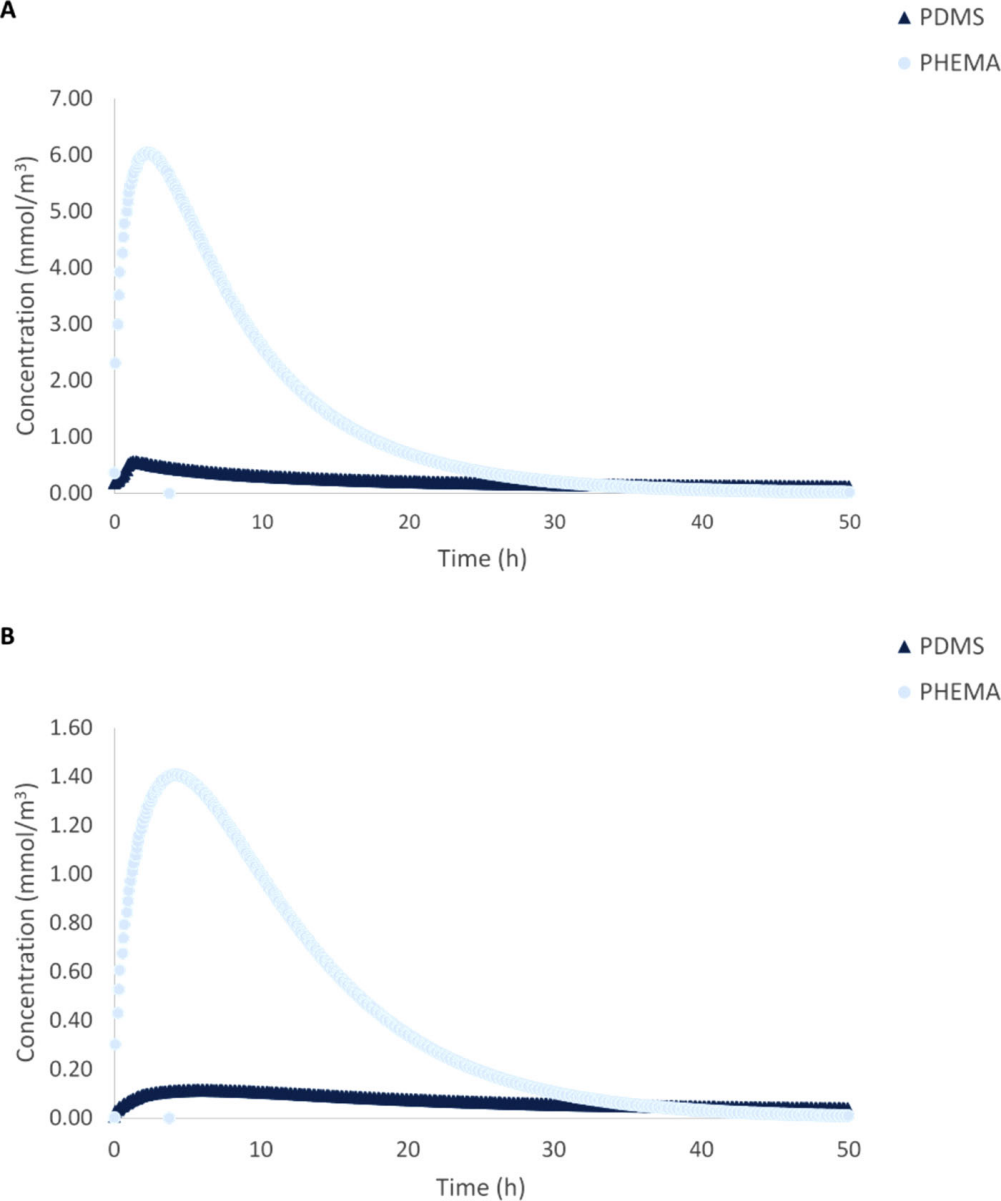
Average dexamethasone concentration in the aqueous (A) and vitreous (B) over a 50-hour period following release from a PDMS IOL and a PHEMA IOL. Maximum concentration (*C_max_*) is represented by the peak on each curve. In the aqueous, *C_max_* values were 5.62 × 10^−4^ mol/m^3^ and 6.04 × 10^−3^ mol/m^3^ at 2.3 h and 1.4 h following release from a PDMS IOL and a PHEMA IOL, respectively. In the vitreous, *C_max_* values were 1.15 × 10^−4^ mol/m^3^ and 1.41 × 10^−3^ mol/m^3^ at 5.9 h and 4.2 h following release from a PDMS IOL and a PHEMA IOL, respectively.

*C_l_* values for dexamethasone following release from both a PHEMA IOL (base case) and a PDMS IOL in each ocular segment are reported in Table 7. *C_l_* was calculated in the period from when the elimination phase began to the end of the study (50 hours). *C_max_* and *t_max_* following release from a PDMS IOL, as well as *C_f_* values (at *t_f_* of 50 hours), utilized in *C_l_* calculations are listed in the Supplementary Materials, Section N. When the IOL material was changed from PHEMA to PDMS, *C_max_* decreased by 89.1% to 91.9% and *C_l_* decreased by 91.3% to 94.6% (least in the sclera, most in the choroid in both cases; see Supplementary Materials, Section N). Concentration profiles of dexamethasone in the vitreous and aqueous following release from both a PDMS IOL and a PHEMA IOL are shown in Figure 6 to illustrate these trends. Decreases in *C_max_* and *C_l_* were observed because dexamethasone diffusion through PDMS is slower than through PHEMA; the diffusion coefficient of dexamethasone in PHEMA is three orders of magnitude greater than in PDMS (diffusion coefficients are provided in Supplementary Materials, Section F). As a result, after 50 hours, only 19.0% of drug was released from the PDMS IOL, whereas more than 99.9% was released from the PHEMA IOL, illustrating that the IOL material itself may be used to modulate release kinetics.

**Table 7.** *C_l_* for Dexamethasone After Release From a PHEMA IOL (Base Case) and PDMS IOL

	*C_l_* (×10^6^ mol/m^3^·h)	
	PHEMA IOL	PDMS IOL
Aqueous	126	9.15
Cornea	122	8.13
Vitreous	30.5	1.70
Retina	8.19	0.461
Sclera	3.14	0.275
Choroid	0.0766	0.00412

*C_l_* was calculated between elimination phase initiation and 50 hours.

## Discussion

Physiologically representative in silico models were generated to characterize IOL drug release to treat post-cataract surgery complications. The ocular temperature profiles from the models matched physiological values, including normal corneal surface and corneal limbus temperatures (Fig. 4; Supplementary Materials, Section J).^42^ The aqueous flow velocity exceeded that of the vitreous because the fluid does not enter the vitreous directly but rather is a result of posterior-directed aqueous flow.^17^^,^^18^ The aqueous vein and choroid were modeled like rigid pipes, which implies continuous drug clearance. However, the literature suggests that flow into aqueous veins is pulsatile^30^ and that a portion of choroidal blood flow is pulsatile,^45^ meaning that clearance may not be continuous. The pulsatile nature of such flows is not well characterized, but models can be updated when physiological or ex vivo verified parameters (frequencies, amplitudes) are obtained.

Restricting flow at different interfaces mimics inflammation and disease. Eliminating flow from the aqueous vein showed that patients with POAG would experience elevated drug concentrations in the sclera and choroid, whereas eliminating flow from the choroid showed that patients with AMD would experience increased drug concentrations in all posterior segments. Adverse effects could occur in both cases. For example, toxic hydroxychloroquine levels may lead to choriocapillaris degeneration,^46^ and elevated amiodarone levels may result in choroidal neovascularization.^47^ Other drugs may also cause adverse side effects in ocular tissue. Choroidal and scleral drug toxicities must be assessed in patients with POAG and posterior tissue toxicity must be analyzed in patients with AMD before an IOL is used for drug delivery. Also, results suggest that, unless AMD is severe (flow reduction greater than 50%), only the drug concentration at the choroid will be altered. The extent of this effect can be fully characterized when a physiological or ex vivo–verified blood flow rate estimate is obtained.

The size and charge of drugs influence their concentration and diffusion through ocular media. In the models, hydrophobic drugs attained higher concentrations than hydrophilic drugs, except where hydrophilic drug molecular size hindered drug diffusion. Restricted diffusion resulted in accumulation and increased concentration, as was observed for dextran in the retina. Clearance depended on molecular size and hydrophilicity/hydrophobicity in all ocular segments; clearance was fastest for hydrophobic small molecules and slowest for hydrophilic macromolecules, trends that are in agreement with results from previous studies.^48^ Dextran thus accumulated in the retina because its large size (40,000 Da) resulted in low retinal pigment epithelium permeability, which is the rate-limiting step.^37^

Delivery profiles are also material specific, illustrating that the material of the IOL can be designed to control delivery. PHEMA released elevated concentrations within a short time frame, whereas PDMS offered sustained delivery for when long delivery times are required. However, to sustain release from PHEMA, loading by supercritical fluid impregnation or external coatings can help to reduce diffusion.^24^ Release times may be further altered by incorporating drugs into the IOL during synthesis at levels above their solubility limits or loading drugs into reservoirs attached to the IOL.^24^ As such, it may be more efficient to control the delivery profile by altering the loading strategy rather than the IOL material. Different forms of loading can be analyzed in models when appropriate diffusion coefficients have been obtained experimentally.

In summary, our results show that diseased conditions lead to drug accumulation in posterior ocular tissue, most notably in the choroid. Accordingly, choroidal drug toxicity should be analyzed prior to use of a drug-eluting IOL in patients with POAG or AMD. As well, drug properties affected maximum concentration and clearance; the highest concentrations and fastest clearance rates were attained by the hydrophobic small molecule, whereas the opposite was true for the hydrophilic macromolecule. Also, the IOL material altered concentration profiles; high concentrations were achieved quickly following release from PHEMA, whereas sustained release was achieved following release from PDMS.

### Research Direction and Future Works

The models developed for the present study offer predictions on drug delivery from the IOL; however, the in silico results require experimental validation in vivo. When specific experimental values have been obtained, the models can be refined and expanded to include a full three-dimensional model of the eye and to take into account buoyant convection,^43^^,^^49^ as well as the effects of gravity,^50^ variations in patient attitude over time (standing, recumbent, supine, prone), the eye being opened or closed, blinking, and head motion. Such models would require considerable computing power but would offer a full depiction of drug distribution in the entire eye.

## Supplementary Material

Supplement 1
