# Supplementary material for: Modeling the Effects of Disease, Drug Properties, and Material on Drug Transport From Intraocular Lenses
Source: Transl Vis Sci Technol. 2022 May 16;11(5):14. doi: 10.1167/tvst.11.5.14 (PMC9123490; doi:10.1167/tvst.11.5.14)
Supplement: Supplement 1 [file tvst-11-5-14_s001.pdf]

Supplementary material

Supplementary Section A- List of abbreviations, acronyms, and symbols

Supplementary Section B- Sub-domain dimensions

Supplementary Section C- Sub-domain material properties

Supplementary Section D- IOL initial concentration selection

Supplementary Section E- Drug diffusion coefficients in ocular tissue

Supplementary Section F- Drug diffusion coefficients in IOL

Supplementary Section G- Time-dependent study details

Supplementary Section H- Drug properties

Supplementary Section I- Data analysis procedure for time-dependent studies

Supplementary Section J- Steady-state temperatures and flow velocities

Supplementary Section K- Elimination removal studies additional information

Supplementary Section L- Flow reduction studies additional information

Supplementary Section M- Drug comparison studies additional information

Supplementary Section N- Material change studies additional information

Supplementary Section O- Background Information on Heat and Mass Transfer Modelling

References

Supplementary Section A- List of abbreviations, acronyms, and symbols

Abbreviation / Symbol	Meaning / Definition
AMD	Age-related macular degeneration
CFD	Computational fluid dynamics
DL	Darcy's law
HEMA	2-hydroxyethyl methacrylate
HT	Heat transfer
IOL	Intraocular lens
LEC	Lens epithelial cell
LF	Laminar flow
MMA	Methyl methacrylate
PCO	Posterior capsule opacification
PDMS	Poly(dimethyl siloxane)
PEA	Phenylethyl acrylate
PHEMA	Poly(2-hydroxyethyl methacrylate)
POAG	Primary open-angle glaucoma
RPE	Retinal pigment epithelium
TDS	Transport of diluted species
B	Body force
C	Time/space dependent model drug concentration
C_b	Bulk drug concentration in surrounding blood
C_f	Final concentration attained at time $\mathrm{t_f}$
C_l	Clearance rate
C_{max}	Maximum concentration
C_p	Specific heat at constant pressure
D_{ij}	Diffusion coefficient of species i and material j
E	Tear evaporation rate
$\mathbf{F}$	Volume force vector
g	Gravitational acceleration
h_1	Convective heat transfer coefficient of the cornea
h_2	Convective heat transfer coefficient of the sclera
$\mathbf{I}$	Identity tensor
i	Subscript denoting modelling sub-domain
J	Diffusive molar flux
k	Permeability
k_s	Mass transfer coefficient of the sclera
k_t	Thermal conductivity
L	Characteristic length (specific to each sub-domain)

**Abbreviation / Symbol**	**Meaning / Definition**
N	Total molar flux
p	Pressure
P_c	Percent change in study result compared to the base case
q	Heat flux
T	Time/space-dependent model temperature
T_a	Ambient temperature
T_{bl}	Blood temperature
t	Time
t_f	Time at which the final concentration (C_f) is attained
t_h	Time for maximum concentration to be reduced by one half
t_{max}	Time to reach maximum concentration
U	Medium flow velocity
$\mathbf{u}$	Velocity vector
u	Transported scalar
x	Thickness of the sclera
X_b	Result of base case study
X_s	Result of study other than the base case
α	Thermal expansion coefficient
Δ	Differential or variation
ε	Corneal surface emissivity
θ	Initial condition (source term)
μ	Dynamic viscosity
ρ	Density
σ	Stefan-Boltzmann constant
τ	Viscous stress tensor
∇	Del operator

Supplementary Section B- Sub-domain dimensions

Dimensions used to build the ocular geometry are listed in Table B-1.

Table B-1: Dimensions used to build ocular geometry.

Sub-domain	Parameter	Value (mm)
1. Cornea	Thickness[1]	0.55
2. Aqueous	Anterior segment depth[2,3,4,*,†]<br>Anterior segment width[1,*]	4.1<br>12
3. Iris	Length[‡]<br>Thickness[5,6]	3.05<br>0.5
4. Ciliary body	Thickness[7]	0.92
5. Sclera	Thickness[1]	1.0
6. Choroid	Thickness[8]	0.25
7. Retina	Thickness[1]	0.25
8. Vitreous	Posterior segment depth[9,§]	16
9. Optic nerve	Thickness[10,‖]	4.0
10. Trabecular meshwork	Length[11]<br>Thickness[11]	0.75<br>0.12
11. Schlemm's canal	Thickness[12]<br>Length[12]	0.050<br>0.25
12. Aqueous vein	Thickness[13]	0.050
13. IOL	Diameter[14]<br>Thickness[14]	6.0<br>1.0
14. Lens capsule	Thickness[1]	0.010

[*]Anterior segment is from posterior cornea (sub-domain 1) to anterior IOL (sub-domain 13).

[†] Anterior chamber depth is known to change after cataract surgery; thus the tabulated value corresponds to the post-cataract surgery dimension. The tabulated value is the average of those reported by references numbered 2, 3, and 4.

[‡] Length was determined by setting pupil length as 4 mm (2 mm in the model half geometry), which is the average of values reported in bright light and in the dark.[15]

[§] Posterior segment is from the IOL centre (sub-domain 13) to posterior sclera (sub-domain 5).

[‖] Half of the optic nerve is included in the model, so thickness was reduced by half.

Supplementary Section C- Sub-domain material properties

Material properties of each sub-domain are listed in Table C-1.

Table C-1: Material properties of each sub-domain.

Sub-domain	Density (kg/m^3)	Thermal conductivity (W/m·K)	Specific heat capacity (J/kg·K)	Dynamic viscosity (Pa·s)*	k/μ (m^2/Pa·s)†
1. Cornea[16,17,18]	1050	0.58	4178	N/A	N/A
2. Aqueous[16,17,18]	1000	0.58	3997	0.00074	N/A
3. Iris[16]	1100	1.680	3650	N/A	N/A
4. Ciliary body[16,‡]	1100	1	3180	N/A	N/A
5. Sclera[16,18,19]	1100	1	3180	N/A	N/A
6. Choroid[17,20,21,22,§]	1060	0.580	3600	0.00035	1.5×10^{-15}
7. Retina[16,17,23]	1000	0.565	3680	N/A	2.4×10^{-15}
8. Vitreous[16,17]	1000	0.594	3997	N/A	8.4×10^{-11}
9. Optic nerve[16, ‡]	1100	1	3180	N/A	N/A
10. Trabecular meshwork[16,‡]	1100	1	3180	N/A	N/A
11. Schlemm's canal[16,‡]	1100	1	3180	N/A	N/A
12. Aqueous vein[20,21,22,§]	1060	0.580	3600	0.00035	N/A
13. IOL[‖]	1000	1	4000	N/A	N/A
14. Lens capsule[‖]	1000	1	4000	N/A	N/A

[*] Only sub-domains involved in the LF interface require dynamic viscosity inputs.

[†] Only sub-domains involved in the DL interface require k/μ inputs, where k is the permeability and μ is the dynamic viscosity of the fluid.

[‡] Assumed to be the same as the sclera.

[§] Values pertaining to blood plasma were used because blood flows through these sub-domains.

[‖] Values were not found in the literature. Varying parameters +/- 50% of tabulated values did not result in a changed temperature profile.

Note that no velocity profile was visible in the transition between segments modelled in the DL interface because the values were relatively small. This is attributed to low values of the quotient of permeability and dynamic viscosity (k/μ) as indicated in the right-hand column of Table C-1, combined with relatively small pressure variations (maximum of 700 Pa, as listed in Table 2 of the main document). According to Darcy's law,[24] instantaneous flow rates (velocity U) correspond to the product of k/μ with a pressure drop Δp over a distance L, such that:

$$U = -\frac{k}{\mu L}\Delta p \quad \text{(C-1)}$$

Thus, assuming the pressure drop to take place over a small distance of 1 mm (e.g., between DL1 and DL2), the largest possible value for instantaneous flow rate is 5.9×10^{-5} m/s (i.e., two orders of magnitude lower than blood velocities listed in Table 2). The pressure change between DL1 and DL2 is equal to a variation from 15 to 9.8 mmHg. Although this is high, it is also reasonable. For example, previous studies of the pressure along the retina find that there can be differences of up to 1.5 mmHg due to slight physical differences along the surfaces.[25] Darcy's law was applied to describe the flow in the retina and choroid adjoining the vitreous, as described in Figure 2. The carrying capacity of the choroidal veins exceeds the low value from the aqueous to vitreous humour on a local and global perspective. The aqueous humour volumetric flow rate is estimated at 2.4 µL/min[26] and the blood velocity at the choroid is 0.75 ml/min, which enables the clearance of excess fluid. Studies indicate that an insignificant contribution of liquid results from metabolism within the vitreous and that the majority of overall flow will be from the aqueous humour.[27] Importantly, analyses from numerous sources summarized by Smith *et al.* (2020) show the high capability for the liquid flow through the vitreous and transported away from vortex veins connected to the choroid, which underlines the importance of observing and estimating such flow within models.[27]

Supplementary Section D- IOL initial concentration selection

Initial drug concentration in the IOL was set by ensuring that solubility limits were not surpassed.

D.1- PHEMA

Because PHEMA is a hydrophilic biomaterial, initial drug concentration was chosen by analyzing the aqueous solubilities of each model drug, which are reported in Table D-1.

Table D-1: Drug aqueous solubilities.

Drug	Mass solubility (g/m^3)	Molar solubility (mol/m^3)
Dexamethasone[28]	89	0.23
Ganciclovir[29]	4300	16.8
Dextran[30]	30000	0.75

Initial drug concentration was set to be less than the lowest drug molar aqueous solubility (dexamethasone). The closest round number to the molar solubility limit was used (0.20 mol/m^3).

D.2- PDMS

Only dexamethasone release from a PDMS IOL was simulated. The solubility of dexamethasone in PDMS oil (viscosity 1000 cSt) was reported as 89 mg/kg,[31] and the viscosity of PDMS oil (viscosity 1000 cSt) is 975 kg/m^3.[32] Using these values, the solubility of dexamethasone in PDMS was calculated to be 86.8 g/m^3, which is equivalent to 0.22 mol/m^3. The initial concentration of dexamethasone in the PDMS IOL was thus set as 0.20 mol/m^3 because:

1. This value is within the calculated solubility limit (it is less than 0.22 mol/m^3).
2. This value is what was set for the studies in which drugs were released from PHEMA. Using the same initial concentration allowed for the effect of IOL material on drug transport to be easily assessed.

Supplementary Section E- Drug diffusion coefficients in ocular tissue

Diffusion coefficients of the three model drugs in each sub-domain are listed in Table E-1. The optic nerve (sub-domain 9) was not incorporated during drug transport studies, and thus is excluded from the list. Sub-domain 13 (IOL) is also excluded because diffusion coefficients are discussed in detail in Supplementary Section F.

Table E-1: Diffusion coefficients of model drugs in each sub-domain. Diffusion coefficients are in $\times 10^{11}$ m^2/s.

Sub-domain	Dexamethasone	Ganciclovir	Dextran
1. Cornea[33,34,35]	4.18[*,p]	2.10[*,r]	0.27[*,r]
2. Aqueous[36,37,38]	720[p]	100[r]	83[p]
3. Iris[33,37,35,*]	11.1[*,p]	5.51[r]	4.3[*,b]
4. Ciliary body[33,37,35,*]	11.1[*,p]	5.51[r]	4.3[*,b]
5. Sclera[33,37,35]	11.1[*,p]	5.51[r]	4.3[*,b]
6. Choroid[33,37,39]	11.4[*,p]	5.51[r]	0.0125[b]
7. Retina[33,37,39]	11.4[*,p]	5.51[r]	0.0125[b]
8. Vitreous[36,37,38]	180[p]	98.9[r]	22[p]
10. Trabecular meshwork[33,37,35,†]	11.1[*,p]	5.51[r]	4.3[*,b]
11. Schlemm's canal[33,37,35,†]	11.1[*,p]	5.51[r]	4.3[*,b]
12. Aqueous vein[36,37,38,‡]	720[p]	100[r]	83[p]
14. Lens capsule[36,37,38,§]	180[p]	98.9[r]	22[p]

[*] Calculated by multiplying reported permeability value by layer thickness.
[†] Assumed to be the same as the sclera.
[‡] Assumed to be the same as the aqueous.
[§] Assumed to be the same as the vitreous.
[p] Reported value is for porcine tissue.
[r] Reported value is for rabbit tissue.
[b] Reported value is for bovine tissue.

Supplementary Section F- Drug diffusion coefficients in IOL

Drug diffusion coefficients in the IOL were obtained by analyzing literature sources.

F.1- PHEMA

The diffusion coefficient of each drug in PHEMA was required because simulations involved release of all drugs from a PHEMA IOL.

F.1.1- Dexamethasone

The diffusion coefficient of dexamethasone in PHEMA was determined by Kim and Chauhan in 2008.[40] PHEMA hydrogels were prepared using ethylene glycol dimethacrylate (EGDMA) as a cross-linker. Gel water volume fraction was 0.42, and cross-linker density was 73 wt%.[40] Under these conditions, the diffusion coefficient of dexamethasone in PHEMA was found to be 1.08×10^{-11} m^2/s.[40]

F.1.2- Ganciclovir

No studies that analyze ganciclovir diffusion in PHEMA were found in the literature. The diffusion coefficient of ganciclovir in PHEMA was thus determined as follows:

$$D_{g,p} = D_{g,w} \times \frac{D_{d,p}}{D_{d,w}} \tag{F-1}$$

where $D_{g,p}$ is the diffusion coefficient of ganciclovir in PHEMA (m^2/s), $D_{g,w}$ is the diffusion coefficient of ganciclovir in water (m^2/s), $D_{d,p}$ is the diffusion coefficient of dexamethasone in PHEMA (m^2/s), and $D_{d,w}$ is the diffusion coefficient of dexamethasone in water (m^2/s). The values of the diffusion coefficient of ganciclovir and dexamethasone in water were reported as 5.11×10^{-10} m^2/s and 4.72×10^{-10} m^2/s respectively.[39] Accordingly, using Equation F-1:

$$D_{g,p} = 5.11 \times 10^{-10}\,\frac{\mathrm{m}^2}{\mathrm{s}} \times \frac{1.08 \times 10^{-1}\,\frac{\mathrm{m}^2}{\mathrm{s}}}{4.72 \times 10^{-10}\,\frac{\mathrm{m}^2}{\mathrm{s}}} = 1.17 \times 10^{-11}\,\frac{\mathrm{m}^2}{\mathrm{s}}$$

F.1.3- Dextran

Liu et al. (2013) analyzed dextran diffusion in a 30 wt% MMA/70 wt% HEMA gel in 2013.[41] The gel was prepared using EGDMA as the crosslinker, and several water volume fractions and crosslinker densities were assessed.[41] A gel with a 0.227 water volume fraction and 1% crosslinker density was most similar to the gel utilized in the dexamethasone study performed by Kim and Chauhan (described in Supplementary Section F.1.1).[40] Liu et al. (2013) analyzed dextrans of molecular weights 4000 Da, 10000 Da, and 20000 Da.[41] There was no clear pattern in reported diffusion coefficients; diffusion coefficients for 4000 Da dextran were the highest, and for 10000 Da dextran were the lowest.[41] As such, extrapolation to determine the diffusion coefficient of 40000 Da dextran was deemed infeasible. The diffusion coefficient of the highest molecular weight dextran (20000 Da), which was reported as 6.17×10^{-12} m^2/s, was thus utilized.[41] This value is a rough estimate, because:

1. The gel was made from MMA and HEMA, whereas diffusion coefficients for other drugs are for gels made solely from HEMA.
2. The dextran analyzed was 20000 Da rather than 40000 Da (which is the molecular weight of the model drug).

Though 6.17×10^{-12} m^2/s was the best estimate found in the literature for the diffusion coefficient of dextran through PHEMA, it can be updated once an appropriate diffusion study is conducted.

F.2- PDMS

Only dexamethasone release from a PDMS IOL was modelled. Accordingly, the diffusion coefficient of dexamethasone in PDMS was required. Gehrke et al. (2015) analyzed dexamethasone diffusion in silicone matrices in 2016. PDMS was made from a MED-6015 silicone preparation kit.[42] The diffusion coefficient of dexamethasone in PDMS was found to be 2.0×10^{-14} m^2/s.[42]

Supplementary Section G- Time-dependent study details

The term "study" is used to describe a specific CFD simulation. Study identifications and details are outlined in Table G-1. The drug and IOL material utilized in each study are recorded in the centre two columns. Steady-state interfaces that were changed during each study are listed in the right column. No changes were made to interfaces that are not listed.

Table G-1: Time dependent study identifications and details.

Study identification	Drug	IOL material	Steady-state interface changes
Base case	Dexamethasone	PHEMA	N/A
Aqueous vein blood flow removal	Dexamethasone	PHEMA	LFC disabled
Choroidal blood flow removal	Dexamethasone	PHEMA	LFB disabled
Aqueous vein and choroidal blood flow removal	Dexamethasone	PHEMA	LFB/LFC disabled
50% choroidal blood flow reduction	Dexamethasone	PHEMA	LFB 50% reduction
75% choroidal blood flow reduction	Dexamethasone	PHEMA	LFB 75% reduction
95% choroidal blood flow reduction	Dexamethasone	PHEMA	LFB 95% reduction
99% choroidal blood flow reduction	Dexamethasone	PHEMA	LFB 99% reduction
Ganciclovir for drug property analysis	Ganciclovir	PHEMA	N/A
Dextran for drug property analysis	Dextran	PHEMA	N/A
PDMS for IOL material analysis	Dexamethasone	PDMS	N/A

Supplementary Section H- Drug properties

Selected properties of the three model drugs that were studied are listed in Table H-1.

Table H-1: Properties of model drugs.

Drug	Octanol/water partition coefficient	Molecular diameter (nm)	Molecular weight (Da)
Dexamethasone[39,43]	68	0.52	392
Ganciclovir[39,44]	0.022	0.45	255
Dextran[39,38]	0.33	4.5	40000

Supplementary Section I- Data analysis procedure for time-dependent studies

For studies analyzing drug properties and IOL materials, drug clearance was analyzed by calculating clearance rates (C_l) in each ocular segment using:

$$C_l = \frac{C_{max} - C_f}{t_f - t_{max}} \quad \text{(I-1)}$$

where C_l is the clearance rate (mol/m^3·h), C_{max} is the maximum concentration (mol/m^3) attained at t_{max} (h), and C_f is the final concentration (mol/m^3) attained at t_f (h). For studies analyzing drug properties, t_f was the time at which IOL concentration was reduced to 0.35% of its initial value (7×10^{-4} mol/m^3) because study period lengths varied for different drugs. For studies analyzing IOL materials, t_f was 50 hours (the end of the study period) because study period length was the same as the base case. In studies where blood flow was removed or reduced, drug clearance was assessed by determining the time for maximum concentration in each ocular segment to be reduced by one half (t_h). Calculations of C_l produced results that were not comparable to the base case because t_{max} was substantially increased in certain ocular segments. C_l, t_h, and C_{max} values obtained in all studies were compared to the base case using:

$$P_c = \frac{X_s - X_b}{X_b} \times 100\% \quad \text{(I-2)}$$

where P_c is the percent change (%), X_s is the result of the study (t_h, C_l, or C_{max}) and X_b is the result of the base case (t_h, C_l, or C_{max}).

Supplementary Section J- Steady-state temperatures and flow velocities

Flow profiles of the aqueous humour, vitreous humour, choroid, and aqueous veins were obtained by CFD modelling. Steady-state temperatures were also recorded. The average and maximum flow velocities attained in each tissue are listed in Table J-1. Average temperatures are listed in Table J-2.

Table J-1: Average and maximum steady-state flow velocities.

Sub-domain	Tissue	Average velocity (m/s)	Maximum velocity (m/s)
2	Aqueous humour	9.63×10^{-6}	5.34×10^{-5}
8	Vitreous humour	6.47×10^{-9}	8.75×10^{-8}
6	Choroid	5.49×10^{-3}	8.68×10^{-3}
12	Aqueous vein	9.59×10^{-3}	1.51×10^{-2}

Table J-2: Average steady-state temperatures.

Location	Average temperature (°C)	Reference values (°C)[19,45]
Corneal central surface	34.5	33.2-35.2
Corneal limbus surface	35.5	33.9-35.5
Sclera outer surface	36.5	35.6-37.1
Blood	37.0	37.0

Temperature results match values previously reported by Purslow et al. (2005) and Gokul et al. (2014).[19,45]

Supplementary Section K- Blood flow removal additional information

C_{max}, t_{max}, and t_h for dexamethasone in each ocular segment for studies in which blood flow was removed are listed in Table K-1. The base case is included for comparison.

Table K-1: Maximum concentration (C_{max}), time to maximum concentration (t_{max}), and time for maximum concentration to be reduced by one half (t_h) for dexamethasone in each ocular segment for blood flow removal and the base case.

**Study identification**	**Base case**	**Aqueous vein blood flow removal**	**Choroidal blood flow removal**	**Aqueous vein and choroidal blood flow removal**
Interfaces disabled	N/A	LFC	LFB	LFB/LFC
	C_{max} **(×10³ mol/m³)**	C_{max} **(×10³ mol/m³)**	C_{max} **(×10³ mol/m³)**	C_{max} **(×10³ mol/m³)**
Aqueous	6.04	5.92	6.22	6.07
Cornea	5.63	5.39	5.87	5.60
Vitreous	1.41	1.43	1.86	1.82
Retina	0.368	0.372	1.55	1.51
Sclera	0.146	0.677	0.999	0.964
Choroid	0.00335	0.0354	1.34	1.30
	t_{max} **(h)**	t_{max} **(h)**	t_{max} **(h)**	t_{max} **(h)**
Aqueous	2.3	2.2	2.5	2.4
Cornea	3.9	3.8	4.2	4.1
Vitreous	4.2	3.9	9.3	8.9
Retina	5.4	5.2	12.3	12
Sclera	3.8	2	12.5	12.3
Choroid	6.7	1.7	13.4	13.1
	t_h **(h)**	t_h **(h)**	t_h **(h)**	t_h **(h)**
Aqueous	6.6	6.5	9.4	9.1
Cornea	6.9	6.8	10.0	9.7
Vitreous	9.3	9.3	37.1	35.5
Retina	9.4	9.4	37.0	35.5
Sclera	6.0	2.1	36.8	35.3
Choroid	9.5	2.2	36.6	35.6

Percent changes in C_{max} and t_h for choroidal blood flow removal compared to aqueous vein and choroidal blood flow removal are listed in Table K-2.

Table K-2: Percent changes in maximum concentration (C_{max}) and time for maximum concentration to be reduced by one half (t_h) for choroidal blood flow removal compared to aqueous vein and choroidal blood flow removal. No percent changes exceed the 5% threshold.

	C_{max} (% change)	t_h (% change)
Aqueous	2.57%	3.30%
Cornea	4.76%	3.09%
Vitreous	2.28%	4.51%
Retina	2.72%	4.23%
Sclera	3.62%	4.25%
Choroid	2.78%	2.81%

Supplementary Section L- Blood flow reduction additional information

Inlet velocities at the LFB2 boundary were altered to reflect the appropriate percentage reduction in choroidal blood flow. Inlet velocities corresponding to each percentage reduction are listed in Table L-1.

Table L-1: Percentage reduction in choroidal blood flow and corresponding inlet velocities.

Percentage reduction (%)	LFB2 inlet velocity ($\times 10^3$ m/s)
50	3.0
75	1.5
95	0.30
99	0.060

C_{max}, t_{max}, and t_h for dexamethasone with reduced choroidal flow in each ocular segment are reported in Table L-2.

Table L-2: Maximum concentration (C_{max}), time to maximum concentration (t_{max}), and time for maximum concentration to be reduced by one half (t_h) for dexamethasone with reduced choroidal blood flow.

Study identification	50% reduction	75% reduction	95% reduction	99% reduction
	C_{max} (×10³ mol/m³)	C_{max} (×10³ mol/m³)	C_{max} (×10³ mol/m³)	C_{max} (×10³ mol/m³)
Aqueous	6.10	6.10	6.12	6.14
Cornea	5.67	5.68	5.71	5.73
Vitreous	1.42	1.42	1.47	1.49
Retina	0.385	0.406	0.484	0.603
Sclera	0.142	0.144	0.167	0.179
Choroid	0.00568	0.0102	0.0399	0.150
	t_{max} (h)	t_{max} (h)	t_{max} (h)	t_{max} (h)
Aqueous	2.3	2.3	2.3	2.3
Cornea	3.9	3.9	3.9	4
Vitreous	4.2	4.3	4.3	4.7
Retina	5.5	5.6	5.9	6.7
Sclera	3.6	3.4	3.1	4.7
Choroid	7.0	6.9	7.6	8.3
	t_h (h)	t_h (h)	t_h (h)	t_h (h)
Aqueous	6.7	6.7	6.9	7.2
Cornea	7.0	7.0	7.2	7.4
Vitreous	9.5	9.6	10.3	11.5
Retina	9.5	9.7	10.3	11.6
Sclera	6.1	6.4	6.5	11.7
Choroid	9.6	10.1	10.2	11.8

Supplementary Section M- Drug property analysis additional information

C_l was calculated between elimination phase initiation and the time at which the IOL concentration was reduced to 0.35% of its initial value using Equation I-1. C_{max} and t_{max} of each drug in each ocular segment are reported in Table 6. The time at which IOL concentration was reduced to 0.35% of its initial value (t_f) was 21.3, 24.9, and 244 hours for dexamethasone, ganciclovir, and dextran, respectively. Concentrations at t_f (C_f) in each ocular segment for each drug are listed in Table M-1.

Table M-1: Concentrations in each ocular segment for each drug when IOL concentration was reduced to 0.35% of its initial value.

	**Dexamethasone**	**Ganciclovir**	**Dextran**
	C_f (×10^3 mol/m^3)	C_f (×10^3 mol/m^3)	C_f (×10^3 mol/m^3)
Aqueous	0.597	0.634	0.609
Cornea	0.706	0.785	0.598
Vitreous	0.294	0.396	0.906
Retina	0.0885	0.123	0.531
Sclera	0.0158	0.0122	0.0111
Choroid	0.000933	0.000680	0.0000492

The effects of drug properties on C_{max} and C_l were assessed by calculating changes for ganciclovir and dextran compared to dexamethasone. Values are listed in Table M-2. Increases greater than 5% are shown in green, and decreases greater than 5% are shown in red.

Table M-2: Percent change in maximum concentration (C_{max}) and clearance rate (C_l) for ganciclovir and dextran compared to dexamethasone. Increases greater than 5% are in green, and decreases greater than 5% are in red.

	**Ganciclovir**	**Dextran**
	**C_{max} (% change)**	**C_{max} (% change)**
Aqueous	1.82	-13.6
Cornea	-22.0	-55.1
Vitreous	-6.07	1.98
Retina	-14.5	111
Sclera	-62.1	-61.3
Choroid	-54.1	-96.0
	**C_l (% change)**	**C_l (% change)**
Aqueous	-15.9	-93.3
Cornea	-35.7	-96.9
Vitreous	-30.3	-96.3
Retina	-35.8	-92.5
Sclera	-68.9	-97.4
Choroid	-69.7	-99.7

Supplementary Section N- IOL material analysis additional information

C_l was calculated between elimination phase initiation and 50 hours using Equation I-1. C_{max} and t_{max} for dexamethasone following release from a PHEMA IOL (base case) are reported in Table 6. C_{max} and t_{max} for dexamethasone following release from a PDMS IOL are listed in Table N-1.

Table N-1: Maximum concentration (C_{max}) and time to maximum concentration (t_{max}) for dexamethasone after release from a PDMS IOL.

	C_{max} (×10^3 mol/m^3)	t_{max} (h)
Aqueous	0.562	1.4
Cornea	0.506	2.6
Vitreous	0.115	5.9
Retina	0.0312	6.4
Sclera	0.0159	2.1
Choroid	0.000271	8.6

When analyzing IOL materials, t_f was 50 hours, and concentrations at t_f for dexamethasone after release from a PHEMA and PDMS IOL are reported in Table N-2.

Table N-2: Concentrations in each ocular segment at 50 hours following release from a PHEMA IOL and a PDMS IOL.

	PHEMA IOL	PDMS IOL
	C_f (×10^3 mol/m^3)	C_f (×10^3 mol/m^3)
Aqueous	0.0185	0.117
Cornea	0.0217	0.121
Vitreous	0.00963	0.0399
Retina	0.00292	0.0111
Sclera	0.000478	0.00273
Choroid	0.0000317	0.000101

To assess the effects of IOL material on C_{max} and C_l, changes for release from a PDMS IOL compared to release from a PHEMA IOL were calculated. All changes listed in Table N-3 are decreases that surpass the 5% threshold, and are thus highlighted in red.

Table N-3: Percent change in maximum concentration (C_{max}) and clearance rate (C_l) for dexamethasone release from a PDMS IOL compared to a PHEMA IOL. All values are decreases that surpass the 5% threshold, and are thus highlighted in red.

	C_{max} (% change)	C_l (% change)
Aqueous	-90.7	-92.8
Cornea	-91.0	-93.3
Vitreous	-91.8	-94.4
Retina	-91.5	-94.4
Sclera	-89.1	-91.3
Choroid	-91.9	-94.6

The percent of drug released from the IOL was calculated using:

$$P_r(t) = \left(1 - \frac{C_t}{C_i}\right) \times 100\% \qquad \text{(N-1)}$$

where $P_r(t)$ is the percent released at time t (%), C_t is the IOL concentration at time t (mol/m^3), and C_i is the initial IOL concentration (mol/m^3). At 50 hours, the IOL concentration was 0.162 mol/m^3 and 2.12×10^{-5} mol/m^3 for release from the PDMS and PHEMA IOL, respectively. In both cases, initial IOL concentration was 0.20 mol/m^3. Using Equation N-1 for PDMS:

$$P_r(t)_{PDMS} = \left(1 - \frac{0.162\,\frac{\text{mol}}{\text{m}^3}}{0.20\,\frac{\text{mol}}{\text{m}^3}}\right) \times 100\% = 19.0\%$$

Using Equation N-1 for PHEMA:

$$P_r(t)_{PHEMA} = \left(1 - \frac{2.12 \times 10^{-5}\,\frac{\text{mol}}{\text{m}^3}}{0.20\,\frac{\text{mol}}{\text{m}^3}}\right) \times 100\% = 99.989\% \approx 100\%$$

Supplementary Section O- Background Information on Heat and Mass Transfer Modelling

The CFD modelling software COMSOL Multiphysics®[46] incorporates equations governing heat and mass transfer and applies these principles to defined modelling domains. The following description is offered as a summary of applied equations and in addition to governing equations for convective and radiative heat transfer presented in Table 1 and for mass transfer presented in Table 3 (main text). Boundary conditions and simplifications are specific to each sub-domain, as described in the main text and in this supplementary information, and in accordance with related published literature.[2,3,16,17,19, 24,36,47,48,49,50]

Conductive heat transfer (Fourier's law)[47] relates the heat flux (q) to a temperature gradient (∇T) through a proportionality constant, (k_t), which is the thermal conductivity specific to each material (described in Table C-1):

$$q = -k_t \nabla T \tag{O-1}$$

Diffusive mass transfer (Fick's first law)[47] relates diffusion flux (J) to a concentration gradient (∇C) through a proportionality constant, (D_{ij}), which is the diffusivity (diffusion coefficient) specific to species i and material j (described in Table E-1):

$$J = -D_{ij} \nabla C \tag{O-2}$$

Changes in concentration over time (Fick's second law) may be expressed as:

$$\frac{\partial C}{\partial t} = -D_{ij} \nabla^2 C \tag{O-3}$$

Along with mass transfer, the momentum and continuity equations are incorporated into the CFD software to model motion and transport within the fluid. These are described in detail in the COMSOL Multiphysics® User's Manual[46] and summarized here for context of modelling

application featuring fluid properties, laminar flow, transport of dilute species, and compressible flow.

The equations of motion for a single-phase fluid are the continuity equation and the momentum equation. For weakly compressible flow, the compressible form of the Navier-Stokes and continuity equations is applied. In their general form, these equations may be stated as:

$$\frac{\partial \rho}{\partial t} + \nabla \cdot (\rho \mathbf{u}) = 0 \tag{O-4}$$

$$\rho \frac{\partial \mathbf{u}}{\partial t} + \rho \mathbf{u} \cdot \nabla \mathbf{u} = -\nabla p + \nabla \cdot \left[\mu (\nabla \mathbf{u} + (\nabla \mathbf{u})^T - \frac{2}{3} \mu (\nabla \cdot \mathbf{u}) \mathbf{I} \right] + \mathbf{F} \tag{O-5}$$

where ρ is the fluid density (as described in Table C-1), **u** is the velocity vector, p is the pressure (Table 2), μ is the fluid dynamic viscosity (Table C-1), T is the fluid temperature (absolute), **I** is the identity tensor, and **F** is the volume force vector. Equation O-4 is the continuity equation and represents conservation of mass. Equation O-5 is a vector equation representing conservation of momentum, in which the term on the left-hand side corresponds to the inertial forces, and the terms on the right-hand side correspond to pressure forces, viscous forces, and external forces applied to the fluid, respectively. Upon simplification, (e.g., for diffusion across a membrane), this equation describes Darcy's law as described in Section C.

For the transport of dilute species, the generic scalar convection-diffusion transport equation may be expressed as:

$$\frac{\partial u}{\partial t} + \mathbf{u} \cdot \nabla u = \nabla \cdot \left(D_{ij} \nabla u \right) + \theta \tag{O-6}$$

where u is a transported scalar (which in our study is the species transport), $\mathbf{u}$ is the convective velocity vector, D_{ij} is the diffusion coefficient of species i in medium j (as listed in Table E-1), and θ is an initial condition (i.e., a source term).

Conservation of Energy was also applied to solve for heat transfer in fluids interface:

$$\rho C_p\left(\frac{\partial T}{\partial t}+\mathbf{u}\cdot\nabla T\right)+\ \nabla\cdot \mathrm{q}=\ \alpha T\left(\frac{\partial p}{\partial t}+\mathbf{u}\cdot\nabla p\right)+\ \tau{:}\nabla\mathbf{u} \qquad (0\text{-}7)$$

where C_p is the specific heat capacity at constant pressure, which is assumed to be the same as that of water at body temperature (i.e., 4.2×10^3 J/(kg·K), assigned from the software property library) [51]; T is the fluid temperature, q is the conductive heat flux (radiation is neglected); α is the coefficient of thermal expansion, which is assumed to be the same as that of water at body temperature (i.e., 3.37×10^{-4} 1/K) [51]; p is the pressure, and τ is the viscous stress tensor. In this equation, energy variations associated with changes in temperature and the net heat flux are balanced by variation associated with changes in pressure as well as viscous dissipation in the fluid. Furthermore, the coefficient of thermal expansion may be expressed as:

$$\alpha =\ -\frac{1}{\rho}\frac{\partial\rho}{\partial T} \qquad (0\text{-}8)$$

Rearranging and integrating this equation, the buoyant convection equations applied in the aqueous humor feature a body force term stemming from the thermal and gravity effects. This body force may be expressed as:

$$B =\ \rho g\alpha(T-T_a) \qquad (0\text{-}9)$$

where g is the gravitational acceleration and T_a is the reference ambient temperature.

References

1. Steinert R, Jain R. Ophthalmologic applications: introduction. In: Ratner B, Hoffman A, Schoen F, Lemons J, eds. *Biomaterials Science- An Introduction to Materials in Medicine*. 3rd ed. Elsevier; 2013:905-956.

2. Kim M, Park KH, Kim T, Kim DM. Changes in anterior chamber configuration after cataract surgery as measured by anterior segment optical coherence tomography. *Korean J Ophthalmol*. 2011;25(2):77-83. doi:10.3341/kjo.2011.25.2.77

3. Lee H, Zukaite I, Juniat V, Dimitry ME, Lewis A, Nanavaty MA. Changes in symmetry of anterior chamber following routine cataract surgery in non-glaucomatous eyes. *Eye Vis*. 2019;6(1). doi:10.1186/s40662-019-0144-3

4. Baxant A, Hornová J. Anterior chamber morphometry before and after cataract surgery. *J Clin Exp Ophthalmol*. 2016;07(02). doi:10.4172/2155-9570.1000548

5. Wang B, Narayanaswamy A, Amerasinghe N, et al. Increased iris thickness and association with primary angle closure glaucoma. *Br J Ophthalmol*. 2011;95(1):46-50. doi:10.1136/bjo.2009.178129

6. Simpson MJ, Muzyka-Woźniak M. Iris characteristics affecting far peripheral vision and negative dysphotopsia. *J Cataract Refract Surg*. 2018;44(4):459-465. doi:10.1016/j.jcrs.2018.01.028

7. Wang Z, Chung C, Lin J, Xu J, Huang J. Quantitative measurements of the ciliary body in eyes with acute primary-angle closure. *Invest Ophthalmol Vis Sci*. 2016;57(7):3299-3305. doi:10.1167/iovs.16-19558

8. Entezari M, Karimi S, Ramezani A, Nikkhah H, Fekri Y, Kheiri B. Choroidal thickness in healthy subjects. *J Ophthalmic Vis Res*. 2018;13(1):39-43. doi:10.4103/jovr.jovr_148_16

9. Hashemi H, Khabazkhoob M, Miraftab M, et al. The distribution of axial length, anterior chamber depth, lens thickness, and vitreous chamber depth in an adult population of Shahroud, Iran. *BMC Ophthalmol*. 2012;12(1):50. doi:10.1186/1471-2415-12-50

10. Newman WD. Measurement of optic nerve sheath diameter by ultrasound: a means of detecting acute raised intracranial pressure in hydrocephalus. *Br J Ophthalmol*. 2002;86(10):1109-1113. doi:10.1136/bjo.86.10.1109

11. Chen Z, Sun J, Li M, et al. Effect of age on the morphologies of the human Schlemm's canal and trabecular meshwork measured with swept-source optical coherence tomography. *Eye*. 2018;32(10):1621-1628. doi:10.1038/s41433-018-0148-6

12. Yan X, Li M, Chen Z, Zhu Y, Song Y, Zhang H. Schlemm's canal and trabecular meshwork in eyes with primary open angle glaucoma: a comparative study using high-frequency ultrasound biomicroscopy. *PLoS One*. 2016;4(11). doi:10.1371/journal.pone.0145824

13. Johnstone M, Martin E, Jamil A. Pulsatile flow into the aqueous veins: Manifestations in normal and glaucomatous eyes. *Exp Eye Res*. 2011;92(5):318-327. doi:10.1016/j.exer.2011.03.011

14. Werner L, Mamalis N. Foldable intraocular lenses. In: Kohnen T, Koch DD, eds. *Cataract and Refractive Surgery*. 1st ed. Springer; 2005:63-84.

15. Spector RH. The Pupils. In: Walker HK, Hall WD, Hurst JW, eds. *Clinical Methods: The History, Physical, and Laboratory Examinations*. 3rd ed. Butterworths; 1990. Accessed October 7, 2020. http://www.ncbi.nlm.nih.gov/books/NBK381/

16. Mirnezami SA, Rajaei Jafarabadi M, Abrishami M. Temperature Distribution Simulation of the Human Eye Exposed to Laser Radiation. *J Lasers Med Sci*. 2013;4(4):175-181.

17. Balachandran R, Barocas V. Computer modeling of drug delivery to the posterior eye: effect of active transport and loss to choroidal blood flow. *Pharm Res*. 2008;25(11):2685-2696. doi:10.1007/s11095-008-9691-3

18. Silveira M, Franca A. Simulation of temperature variations in the human eye affected by the presence of a tumor. *Adv Mat Res*. 2013;815:707-711. doi:10.4028/www.scientific.net/AMR.816-817.707

19. Gokul K, Gurung D, Adhikary P. Thermal effects of eyelid in human eye temperature model. *J Appl Math Informatics*. 2014;32(5):649-663. doi:10.14317/jami.2014.649

20. Jiang Y, Zhang J, Zhao W. Effects of the inlet conditions and blood models on accurate prediction of hemodynamics in the stented coronary arteries. *AIP Advances*. 2015;5(5). doi:10.1063/1.4919937

21. Tissue properties- thermal conductivity. IT'IS Foundation. Accessed February 14, 2021. https://itis.swiss/virtual-population/tissue-properties/database/heat-capacity/

22. Tissue properties- heat capacity. IT'IS Foundation. Accessed February 14, 2021. https://itis.swiss/virtual-population/tissue-properties/database/heat-capacity/

23. Semenyuk V. Thermal interaction of multi-pulse laser beam with eye tissue during retinal photocoagulation: analytical approach. *Int J Heat Mass Transf*. 2017;112:480-488. doi:10.1016/j.ijheatmasstransfer.2017.05.013

24. Xu J, Heys J, Barocas V, Randolph T. Permeability and diffusion in vitreous humor: implications for drug delivery. *Pharmaceutical research*. 2000;17(6):664-669. doi:10.1023/A:1007517912927

25. Cakmak I, Ozer A. Pressure gradient in the eye. *New Frontiers in Ophthalmology*. 2018;4. doi:10.15761/NFO.1000213

26. Zhang F, Chen HB, Huang Y. Computer modeling of drug delivery in the anterior human eye after subconjunctival and episcleral implantation. *Computers in biology and medicine*. 2017;89:162-169.

27. Smith DW, Lee CJ, Gardiner BS. No flow through the vitreous humor: How strong is the evidence? *Progress in Retinal and Eye Research*. 2020;78:100845. doi:https://doi.org/10.1016/j.preteyeres.2020.100845

28. O'Neil M. *The Merck Index- An Encyclopedia of Chemicals, Drugs, and Biologicals*. Royal Society of Chemistry; 2013.

29. Yallowsky S, Yan H. *Handbook of Aqueous Solubility Data: An Extensive Compilation of Aqueous Solubility Data for Organic Compounds*. CRC Press; 2003.

30. Dextran Product Information. Sigma-Aldrich. Accessed March 1, 2021. https://www.sigmaaldrich.com/content/dam/sigma-aldrich/docs/Sigma/Product_Information_Sheet/d4626pis.pdf

31. Wrzeszcz A, Dittrich B, Haamann D, et al. Dexamethasone released from cochlear implant coatings combined with a protein repellent hydrogel layer inhibits fibroblast proliferation. *J Biomed Res A*. 2014;102(2):442-454. doi:10.1002/jbm.a.34719

32. PDMS 1000. Sigma-Aldrich. Accessed February 21, 2021. https://www.sigmaaldrich.com/catalog/product/sigma/93880

33. Loch C, Zakelj S, Kristl A, et al. Determination of permeability coefficients of ophthalmic drugs through different layers of porcine, rabbit and bovine eyes. *Eur J Pharm Sci*. 2012;47(1):131-138. doi:10.1016/j.ejps.2012.05.007

34. Tirucherai GS, Dias C, Mitra AK. Corneal permeation of ganciclovir: mechanism of ganciclovir permeation enhancement by acyl ester prodrug design. *J Ocul Pharmacol Ther*. 2002;18(6):535-548. doi:10.1089/108076802321021081

35. Prausnitz MR, Noonan JS. Permeability of cornea, sclera, and conjunctiva: a literature analysis for drug delivery to the eye. *J Pharm Sci*. 1998;87(12):1479-1488. doi:10.1021/js9802594

36. Gisladottir S, Loftsson T, Stefansson E. Diffusion characteristics of vitreous humour and saline solution follow the Stokes Einstein equation. *Graefes Arch Clin Exp Ophthalmol*. 2009;247(12):1677-1684. doi:10.1007/s00417-009-1141-3

37. Tojo K, Nakagawa K, Morita Y, Ohtori A. A pharmacokinetic model of intravitreal delivery of ganciclovir. *Eur J Pharm Biopharm*. 1999;47(2):99-104. doi:10.1016/S0939-6411(98)00073-3

38. Gajraj RTC. *A Study of Drug Transport in the Vitreous Humor: Effect of Drug Size; Comparing Micro- and Macro-Scale Diffusion; Assessing Vitreous Models; and Obtaining in Vivo Data*. University of Toronto; 2012.

39. Del Amo EM, Rimpelä AK, Heikkinen E, et al. Pharmacokinetic aspects of retinal drug delivery. *Prog Retin Eye Res*. 2017;57:134-185. doi:10.1016/j.preteyeres.2016.12.001

40. Kim J, Chauhan A. Dexamethasone transport and ocular delivery from poly(hydroxyethyl methacrylate) gels. *Int J Pharm*. 2008;353(1):205-222. doi:10.1016/j.ijpharm.2007.11.049

41. Liu DE, Kotsmar C, Nguyen F, et al. Macromolecule Sorption and Diffusion in HEMA/MAA Hydrogels. *Ind Eng Chem Res*. 2013;52(50):18109-18120. doi:10.1021/ie402148u

42. Gehrke M, Sircoglou J, Vincent C, Siepmann J, Siepmann F. How to adjust dexamethasone mobility in silicone matrices: a quantitative treatment. *Eur J Pharm Biopharm*. 2015;100. doi:10.1016/j.ejpb.2015.11.018

43. Hansch C, Leo A, Hoekman D. *Exploring QSAR: Hydrophobic, Electronic and Steric Constants*. Vol 2. American Chemical Society; 1995.

44. Sangster J. Octanol-water partition coefficients of simple organic compounds. *J Phys Chem Ref Data*. 1989;18(3):1111-1229. doi:10.1063/1.555833

45. Purslow C, Wolffsohn JS. Ocular surface temperature: a review. *Eye Contact Lens*. 2005;31(3):117-123. doi:10.1097/01.ICL.0000141921.80061.17

46. COMSOL. COMSOL Multiphysics reference manual. Published 2019. Accessed October 30, 2020. https://doc.comsol.com/5.4/doc/com.comsol.help.comsol/COMSOL_ReferenceManual.pdf

47. Incropera F, Dewitt D, Bergman T, Lavine A. *Fundamentals of Heat and Mass Transfer*. 6th ed. John Wiley & Sons; 2007.

48. Penkova A, Moats R, Humayun M, Fraser S, Sadhal S. Diffusive Transport in the Vitreous Humor: Experimental and Analytical Studies. *ASME J Heat Transfer*. 2019;141(5):050801-1-11. doi:10.1115/1.4042297

49. Rattanakijsuntorn K, Penkova A, Sadhal S. Mass diffusion coefficient measurement for vitreous humor using FEM and MRI. 8th TSME – International Conference on Mechanical Engineering. In: *8th TSME – International Conference on Mechanical Engineering*. Vol 297. ; 2018:012024. doi:doi:10.1088/1757-899X/297/1/012024

50. Shafahi M, Vafai K. Human Eye Response to Thermal Disturbances. *J Heat Transfer*. 2011;133(1):011009. doi:10.1115/1.4002360

51. Heys JJ, Barocas VH. A Boussinesq Model of Natural Convection in the Human Eye and the Formation of Krukenberg’s Spindle. *Annals of Biomedical Engineering*. 2002;30(3):392-401. doi:10.1114/1.1477447
